# Versatile stabilized finite element formulations for nearly and fully incompressible solid mechanics

**DOI:** 10.1007/s00466-019-01760-w

**Published:** 2019-09-11

**Authors:** Elias Karabelas, Gundolf Haase, Gernot Plank, Christoph M. Augustin

**Affiliations:** 1 Institute for Mathematics and Scientific Computing, NAWI Graz, University of Graz, Graz, Austria; 2 Gottfried Schatz Research Center: Division of Biophysics, Medical University of Graz, Graz, Austria; 3 BioTechMed-Graz, Graz, Austria; 4 Department of Mechanical Engineering, University of California, Berkeley, Berkeley, CA USA; 5 Present Address: Gottfried Schatz Research Center: Division of Biophysics, Medical University of Graz, Neue Stiftingtalstraße 6 (MC1.D.)/IV, 8010 Graz, Austria

**Keywords:** Incompressible elasticity, Large strain elasticity, Mixed finite elements, Piecewise linear interpolation, Transient dynamics

## Abstract

Computational formulations for large strain, polyconvex, nearly incompressible elasticity have been extensively studied, but research on enhancing solution schemes that offer better tradeoffs between accuracy, robustness, and computational efficiency remains to be highly relevant. In this paper, we present two methods to overcome locking phenomena, one based on a displacement-pressure formulation using a stable finite element pairing with bubble functions, and another one using a simple pressure-projection stabilized $\mathbb {P}_1 - \mathbb {P}_1$ finite element pair. A key advantage is the versatility of the proposed methods: with minor adjustments they are applicable to all kinds of finite elements and generalize easily to transient dynamics. The proposed methods are compared to and verified with standard benchmarks previously reported in the literature. Benchmark results demonstrate that both approaches provide a robust and computationally efficient way of simulating nearly and fully incompressible materials.

## Introduction

Locking phenomena, caused by ill-conditioned global stiffness matrices in finite element analyses, are an often observed and extensively studied issue when modeling nearly incompressible, hyperelastic materials [10, 18, 46, 84, 87]. Typically, methods based on Lagrange multipliers are applied to enforce incompressibility. A common approach is the split of the deformation gradient into a volumetric and an isochoric part [38]. Here, locking commonly arises when unstable standard displacement formulations are used that rely on linear shape functions to approximate the displacement field ${\MakeLowercase {\mathbf {u}}}$ and piecewise-constant finite elements combined with static condensation of the hydrostatic pressure $p$, e.g., $\mathbb {P}_1 - \mathbb {P}_0$ elements. It is well known that in such cases solution algorithms may exhibit very low convergence rates and that variables of interest such as stresses can be inaccurate [41].

From mathematical theory it is well known that approximation spaces for the primal variable ${\MakeLowercase {\mathbf {u}}}$ and $p$ have to be well chosen to fulfill the Ladyzhenskaya–Babuŝka–Brezzi (LBB) or *inf–sup* condition [9, 19, 26] to guarantee stability. A classical stable approximation pair is the Taylor–Hood element [78], however, this requires quadratic ansatz functions for the displacement part. For certain types of problems higher order interpolations can improve efficiency as higher accuracy is already reached with coarser discretizations [25, 57]. In many applications though, where geometries are fitted to, e.g., capture fine structural features, this is not beneficial due to a possible increase in degrees of freedom and consequently a higher computational burden. Also for coupled problems such as electromechanical or fluid–structure–interaction models high-resolution grids for mechanical problems are sometimes required when interpolations between grids are not desired [5, 51]. As a remedy for these kind of applications quasi Taylor–Hood elements with an order of $\tfrac{3}{2}$ have been considered, see [62], as well as equal order linear pairs of ansatz functions which has been a field of intensive research in the last decades, see [6, 48] and references therein. Unfortunately, equal order pairings do not fulfill the LBB conditions and hence a stabilization of the element is of crucial importance. There is a significant body of literature devoted to stabilized finite elements for the Stokes and Navier–Stokes equations. Many of those methods were extended to incompressible elasticity, amongst other approaches by Hughes, Franca, Balestra, and collaborators [39, 47]. Masud and co-authors followed an idea by means of variational multiscale (VMS) methods [58–60, 85], a technique that was recently extended to dynamic problems (D-VMS) [66, 71]. Further stabilizations of equal order finite elements include orthogonal sub-scale methods [24, 27, 30, 54] and methods based on pressure projections [33, 86]. Different classes of methods to avoid locking for nearly incompressible elasticity were conceived by introducing nonconforming finite elements such as the Crouzeix–Raviart element [32, 37] and Discontinuous Galerkin methods [49, 80]. Enhanced strain formulations [64, 79] have been considered as well as formulations based on multi-field variational principles [17, 68, 69].

In this study we introduce a novel variant of the MINI element for accurately solving nearly and fully incompressible elasticity problems. The MINI element was originally established for computational fluid dynamics problems [3] and pure tetrahedral meshes and previously used in the large strain regime, e.g. in [25, 56]. We extend the MINI element definition for hexahedral meshes by introducing two bubble functions in the element and provide a novel proof of stability and well-posedness in the case of linear elasticity. The support of the bubble functions is restricted to the element and can thus be eliminated from the system using static condensation. This also allows for a straightforward inclusion in combination with existing finite element codes since all required implementations are purely on the element level. Additionally, we introduce a pressure-projection stabilization method originally published for the Stokes equations [14, 33] and previously used for large strain nearly incompressible elasticity in the field of particle finite element methods and plasticity [22, 65]. Due to its *simplicity*, this type of stabilization is especially attractive from an implementation point of view.

Robustness and performance of both the MINI element and the pressure-projection approach are verified and compared to standard benchmarks reported previously in literature. A key advantage of the proposed methods is their *high versatility*: first, they are readily applicable to nearly and fully incompressible solid mechanics; second, with little adjustments the stabilization techniques can be applied to all kinds of finite elements, in this study we investigate the performance for hexahedral and tetrahedral meshes; and third, the methods generalize easily to transient dynamics.

Real world applications often require highly-resolved meshes and thus efficient and massively parallel solution algorithms for the linearized system of equations become an important factor to deal with the resulting computational load. We solve the arising saddle-point systems by using a GMRES method with a block preconditioner based on an algebraic multigrid (AMG) approach. Extending our previous implementations [5] we performed the numerical simulations with the software *Cardiac Arrhythmia Research Package* (CARP) [82] which relies on the MPI based library *PETSc* [12] and the incorporated solver suite *hypre/BoomerAMG* [43]. The combination of these advanced solving algorithms with the proposed stable elements which only rely on linear shape functions proves to be *very efficient* and renders feasible simulations on grids with high structural detail.

The paper is outlined as follows: Sect. 2 summarizes in brief the background on the methods. In Sect. 3, we introduce the finite element discretization and discuss stability. Subsequently, Sect. 4 documents benchmark problems where our proposed elements are applied and compared to results published in the literature. Finally, Sect. 5 concludes the paper with a discussion of the results and a brief summary.

## Continuum mechanics

### Nearly incompressible nonlinear elasticity

Let $\varOmega _0 \subset \mathbb {R}^3$ denote the reference configuration and let $\varOmega _t \subset \mathbb {R}^3$ denote the current configuration of the domain of interest. Assume that the boundary of $\varOmega _0$ is decomposed into $\partial \varOmega _0 = \varGamma _{\mathrm {D},0} \cup \varGamma _{\mathrm {N},0}$ with $|\varGamma _{D,0}| > 0$. Here, $\varGamma _{\mathrm {D},0}$ describes the Dirichlet part of the boundary and $\varGamma _{\mathrm {N},0}$ describes the Neumann part of the boundary, respectively. Further, let ${\MakeLowercase {\mathbf {n}}}_{0}$ be the unit outward normal on $\partial \varOmega _0$. The nonlinear mapping $\varvec{\phi }:\varvec{\mathbf {X}} \in \varOmega _0 \rightarrow {\MakeLowercase {\mathbf {x}}} \in \varOmega _t$, defined by $\varvec{\phi }:= \varvec{\mathbf {X}} + {\MakeLowercase {\mathbf {u}}}(\varvec{\mathbf {X}},t)$, with displacement ${\MakeLowercase {\mathbf {u}}}$, maps points in the reference configuration to points in the current configuration. Following standard notation we introduce the *deformation gradient*$\varvec{F}$ and the Jacobian *J* as

$$\begin{aligned} \varvec{F} := {\text {Grad}}\,\varvec{\phi }= \varvec{I} + {\text {Grad}}\,{\MakeLowercase {\mathbf {u}}},\quad J := \det (\varvec{F}), \end{aligned}$$

and the *left Cauchy–Green tensor* as $\varvec{C} := \varvec{F}^\top \varvec{F}$. Here, ${\text {Grad}}\,(\bullet )$ denotes the gradient with respect to the reference coordinates $\underline{X} \in \varOmega _0$. The displacement field ${\MakeLowercase {\mathbf {u}}}$ is sought as infimizer of the functional

1 $$\begin{aligned} \varPi ^\mathrm {tot}({\MakeLowercase {\mathbf {u}}})&:= \varPi ({\MakeLowercase {\mathbf {u}}}) + \varPi ^\mathrm {ext}({\MakeLowercase {\mathbf {u}}}), \,\,\,\varPi ({\MakeLowercase {\mathbf {u}}}) := \int \limits _{\varOmega _0} \varPsi (\varvec{F}({\MakeLowercase {\mathbf {u}}}))\,\mathrm {d}\varvec{\mathbf {X}},\nonumber \\ \varPi ^{\mathrm {ext}}({\MakeLowercase {\mathbf {u}}})&:= - \rho _0\int \limits _{\varOmega _0} {\MakeLowercase {\mathbf {f}}}({\MakeLowercase {\mathbf {X}}}) \cdot {\MakeLowercase {\mathbf {u}}}\,\mathrm {d}\varvec{\mathbf {X}}- \int \limits _{\varGamma _\mathrm {N,0}} {\MakeLowercase {\mathbf {h}}}({\MakeLowercase {\mathbf {X}}}) \cdot {\MakeLowercase {\mathbf {u}}}\,\mathrm {d}s_{\varvec{\mathbf {X}}}, \end{aligned}$$

over all admissible fields ${\MakeLowercase {\mathbf {u}}}$ with ${\MakeLowercase {\mathbf {u}}} = {\MakeLowercase {\mathbf {g}}}_\mathrm {D}$ on $\varGamma _\mathrm {D,0}$, where, $\varPsi$ denotes the strain energy function; $\rho _0$ denotes the material density in reference configuration; ${\MakeLowercase {\mathbf {f}}}$ denotes a volumetric body force; ${\MakeLowercase {\mathbf {g}}}_\mathrm {D}$ denotes a given boundary displacement; and ${\MakeLowercase {\mathbf {h}}}$ denotes a given surface traction. For ease of presentation it is assumed that $\rho _0$ is constant and ${\MakeLowercase {\mathbf {f}}}$, ${\MakeLowercase {\mathbf {g}}}_\mathrm {D}$, and ${\MakeLowercase {\mathbf {h}}}$ do not depend on ${\MakeLowercase {\mathbf {u}}}$. Existence of infimizers is, under suitable assumptions, guaranteed by the pioneering works of Ball, see [13].

In this study we consider nearly incompressible materials, meaning that $J \approx 1$. A possibility to model this behavior was originally proposed by Flory [38] using a split of the deformation gradient $\varvec{F}$ such that

2 $$\begin{aligned} \varvec{F} = \varvec{F}_\mathrm {vol} \overline{\varvec{F}}. \end{aligned}$$

Here, $\varvec{F}_\mathrm {vol}$ describes the volumetric change while $\overline{\varvec{F}}$ describes the isochoric change. By setting $\varvec{F}_\mathrm {vol} := J^{\frac{1}{3}} \varvec{I}$ and $\overline{\varvec{F}} := J^{-\frac{1}{3}} \varvec{F}$ we get $\det (\overline{\varvec{F}}) = 1$ and $\det (\varvec{F}_\mathrm {vol}) = J$. Analogously, by setting $\varvec{C}_\mathrm {vol} := J^{\frac{2}{3}} \varvec{I}$ and $\overline{\varvec{C}} := J^{-\frac{2}{3}} \varvec{C}$, we have $\varvec{C} = \varvec{C}_\mathrm {vol} \overline{\varvec{C}}$. Assuming a hyperelastic material, the Flory split also postulates an additive decomposition of the strain energy function

3 $$\begin{aligned} \varPsi = \varPsi (\varvec{C}) = \overline{\varPsi }(\overline{\varvec{C}}) + \kappa U(J), \end{aligned}$$

where $\kappa$ is the *bulk modulus*. The function $U(J)$ acts as a penalization of incompressibility and we require that it is strictly convex and twice continuously differentiable. Additionally, a constitutive model for $U(J)$ should fulfill that (i) it vanishes in the reference configuration and that (ii) an infinite amount of energy is required to shrink the body to a point or to expand it indefinitely, i.e.,

$$\begin{aligned} \text{(i) }\ U(1) = 0,\ \text{(ii) } \lim _{J\rightarrow 0+} U(J) = \infty ,\ \lim _{J\rightarrow \infty } U(J) = \infty . \end{aligned}$$

For the remainder of this work we will focus on functions $U(J)$ that can be written as

$$\begin{aligned} U(J) := \frac{1}{2}{(\varTheta (J))}^2. \end{aligned}$$

In the literature many different choices for the function $\varTheta (J)$ are proposed, see, e.g., [34, 42, 66] for examples and related discussion.

As we also want to study the case of full incompressibility, meaning $\kappa \rightarrow \infty$, we need a reformulation of the system. In this work we will use a perturbed Lagrange-multiplier functional, see [4, 21, 77] for details, and we introduce

$$\begin{aligned} \varPi ^\mathrm {PL}({\MakeLowercase {\mathbf {u}}}, q) := \int \limits _{\varOmega _0}\overline{\varPsi }(\overline{\varvec{C}}({\MakeLowercase {\mathbf {u}}})) + q \varTheta (J({\MakeLowercase {\mathbf {u}}})) - \frac{1}{2 \kappa } q^2\,\mathrm {d}\varvec{\mathbf {X}}. \end{aligned}$$

We will now seek infimizers $({\MakeLowercase {\mathbf {u}}}, p) \in V_{{\MakeLowercase {\mathbf {g}}}_\mathrm {D}} \times Q$ of the modified functional

4 $$\begin{aligned} \varPi ^{\mathrm {tot}}({\MakeLowercase {\mathbf {u}}}, q) := \varPi ^\mathrm {PL}({\MakeLowercase {\mathbf {u}}}, q) + \varPi ^{\mathrm {ext}}({\MakeLowercase {\mathbf {u}}}). \end{aligned}$$

To guarantee that the discretization of (4) is well defined, we assume that

$$\begin{aligned} V_{{\MakeLowercase {\mathbf {g}}}_\mathrm {D}} = \{ {\MakeLowercase {\mathbf {v}}} \in {[H^1(\varOmega _0)]}^3 : \left. {\MakeLowercase {\mathbf {v}}}\right| _{\varGamma _{\mathrm {D},0}} = {\MakeLowercase {\mathbf {g}}}_\mathrm {D}\}, \end{aligned}$$

with $H^1(\varOmega _0)$ being the standard Sobolev space consisting of all square integrable functions with square integrable gradient, and $Q=L^2(\varOmega _0)$. Existence of infimizers in $V_{{\MakeLowercase {\mathbf {g}}}_\mathrm {D}}$ cannot be guaranteed in general. However, assuming suitable growth conditions on the strain energy function $\varPsi$, and assuming that the initial data keeps the material in the hyperelastic range, one can conclude that the space $V$ for the infimizer ${\MakeLowercase {\mathbf {u}}}$ contains $V_{{\MakeLowercase {\mathbf {g}}}_\mathrm {D}}$ as a subset, see [13] for details.

To solve for the infimizers of (4) we require to compute the variations of (4) with respect to $\varDelta {\MakeLowercase {\mathbf {u}}}$ and $\varDelta p$

5 $$\begin{aligned} D_{\varDelta {\MakeLowercase {\mathbf {v}}}} \varPi ^\mathrm {PL}({\MakeLowercase {\mathbf {u}}},p)&= \int \limits _{\varOmega _0} \left( \varvec{S}_\mathrm {isc} + p \varvec{S}_\mathrm {vol}\right) :\varvec{\varSigma }({\MakeLowercase {\mathbf {u}}}, \varDelta {\MakeLowercase {\mathbf {v}}}) \,\mathrm {d}\varvec{\mathbf {X}}\nonumber \\&\quad -\,\rho _0\int \limits _{\varOmega _0} {\MakeLowercase {\mathbf {f}}} \cdot \varDelta {\MakeLowercase {\mathbf {v}}}\,\mathrm {d}\varvec{\mathbf {X}}- \int \limits _{\varGamma _{\mathrm {N},0}} {\MakeLowercase {\mathbf {h}}} \cdot \varDelta {\MakeLowercase {\mathbf {v}}}\,\mathrm {d}s_{\varvec{\mathbf {X}}},\nonumber \\ \end{aligned}$$

6 $$\begin{aligned} D_{\varDelta q} \varPi ^\mathrm {PL}({\MakeLowercase {\mathbf {u}}},p)&= \int \limits _{\varOmega _0} \left( \varTheta (J) - \frac{1}{\kappa } p\right) \varDelta q\,\mathrm {d}\varvec{\mathbf {X}}, \end{aligned}$$

with abbreviations as, e.g., in [45]

7 $$\begin{aligned} \varvec{S}_\mathrm {isc}&:= J^{-\frac{2}{3}} \mathrm {Dev}(\overline{\varvec{S}}), \quad \text {where}~\overline{\varvec{S}} := \frac{\partial \overline{\varPsi }(\overline{\varvec{C}})}{\partial \overline{\varvec{C}}} \end{aligned}$$

8 $$\begin{aligned} \varvec{S}_\mathrm {vol}&:= \pi (J) \varvec{C}^{-1}, \quad \text {with}~ \pi (J) := J\varTheta '(J),\end{aligned}$$

9 $$\begin{aligned} \varvec{\varSigma }({\MakeLowercase {\mathbf {u}}}, {\MakeLowercase {\mathbf {v}}})&:= \mathrm {sym}(\varvec{F}^\top ({\MakeLowercase {\mathbf {u}}}) {\text {Grad}}\,{\MakeLowercase {\mathbf {v}}}). \end{aligned}$$

Next, with notations

10 $$\begin{aligned} a_{\mathrm {isc}}({\MakeLowercase {\mathbf {u}}};\varDelta {\MakeLowercase {\mathbf {v}}})&:= \int \limits _{\varOmega _0} \varvec{S}_\mathrm {isc}({\MakeLowercase {\mathbf {u}}}) : \varvec{\varSigma }({\MakeLowercase {\mathbf {u}}},\varDelta {\MakeLowercase {\mathbf {v}}})\,\mathrm {d}\varvec{\mathbf {X}},\end{aligned}$$

11 $$\begin{aligned} a_{\mathrm {vol}}({\MakeLowercase {\mathbf {u}}}, p;\varDelta {\MakeLowercase {\mathbf {v}}})&:= \int \limits _{\varOmega _0} p \varvec{S}_\mathrm {vol}({\MakeLowercase {\mathbf {u}}}) : \varvec{\varSigma }({\MakeLowercase {\mathbf {u}}},\varDelta {\MakeLowercase {\mathbf {v}}})\,\mathrm {d}\varvec{\mathbf {X}},\end{aligned}$$

12 $$\begin{aligned} b_{\mathrm {vol}}({\MakeLowercase {\mathbf {u}}}; \varDelta q)&:= \int \limits _{\varOmega _0} \varTheta (J({\MakeLowercase {\mathbf {u}}})) \varDelta q\,\mathrm {d}\varvec{\mathbf {X}},\end{aligned}$$

13 $$\begin{aligned} c(p,\varDelta q)&:= \frac{1}{\kappa }\int \limits _{\varOmega _0}p \varDelta q\,\mathrm {d}\varvec{\mathbf {X}},\end{aligned}$$

14 $$\begin{aligned} l_\mathrm {body}(\varDelta {\MakeLowercase {\mathbf {v}}})&:= \rho _0\int \limits _{\varOmega _0} {\MakeLowercase {\mathbf {f}}} \cdot \varDelta {\MakeLowercase {\mathbf {v}}}\,\mathrm {d}\varvec{\mathbf {X}},\end{aligned}$$

15 $$\begin{aligned} l_\mathrm {surface}(\varDelta {\MakeLowercase {\mathbf {v}}})&:= \int \limits _{\varGamma _{\mathrm {N},0}} {\MakeLowercase {\mathbf {h}}} \cdot \varDelta {\MakeLowercase {\mathbf {v}}} \,\mathrm {d}s_{\varvec{\mathbf {X}}},\end{aligned}$$

16 $$\begin{aligned} R_\mathrm {upper}({\MakeLowercase {\mathbf {u}}}, p;\varDelta {\MakeLowercase {\mathbf {v}}})&:= a_{\mathrm {isc}}({\MakeLowercase {\mathbf {u}}};\varDelta {\MakeLowercase {\mathbf {v}}}) + a_{\mathrm {vol}}({\MakeLowercase {\mathbf {u}}}, p;\varDelta {\MakeLowercase {\mathbf {v}}}) \nonumber \\&\qquad -\,l_\mathrm {body}(\varDelta {\MakeLowercase {\mathbf {v}}})-l_\mathrm {surface}(\varDelta {\MakeLowercase {\mathbf {v}}}),\end{aligned}$$

17 $$\begin{aligned} R_\mathrm {lower}({\MakeLowercase {\mathbf {u}}}, p;\varDelta q)&:= b_{\mathrm {vol}}({\MakeLowercase {\mathbf {u}}}; \varDelta q) - c(p,\varDelta q), \end{aligned}$$

we formulate the mixed boundary value problem of nearly incompressible nonlinear elasticity via a nonlinear system of equations. This yields a nonlinear saddle-point problem, find $({\MakeLowercase {\mathbf {u}}}, p) \in V_{{\MakeLowercase {\mathbf {g}}}_D} \times Q$ such that

18 $$\begin{aligned} R_\mathrm {upper}({\MakeLowercase {\mathbf {u}}}, p;\varDelta {\MakeLowercase {\mathbf {v}}})&= 0, \end{aligned}$$

19 $$\begin{aligned} R_\mathrm {lower}({\MakeLowercase {\mathbf {u}}}, p;\varDelta q)&=0, \end{aligned}$$

for all $$(\varDelta {\MakeLowercase {\mathbf {v}}}, \varDelta q) \in V_{{\MakeLowercase {\mathbf {0}}}} \times Q$$.

### Consistent linearization

To solve the nonlinear variational Eqs. (18) and (19), with a finite element approach we first apply a Newton–Raphson scheme, for details we refer to [31]. Given a nonlinear and continuously differentiable operator $F:X\rightarrow Y$ a solution to $F(x)=0$ can be approximated by

$$\begin{aligned} x^{k+1}&= x^{k} + \varDelta x, \\ \left. \frac{\partial F}{\partial x}\right| _{x = x^k} \varDelta x&= -F(x^k), \end{aligned}$$

which is looped until convergence. In our case, we have $X = V_{{\MakeLowercase {\mathbf {g}}}_D} \times Q$, $Y = \mathbb {R}^2$, $\varDelta x = {(\varDelta {\MakeLowercase {\mathbf {u}}}, \varDelta p)}^\top$, $x^k = {({\MakeLowercase {\mathbf {u}}}^k, p^k)}^\top$, and $F = {(R_\mathrm {upper}, R_\mathrm {lower})}^\top$. We obtain the following linear saddle-point problem for each $({\MakeLowercase {\mathbf {u}}}^k, p^k) \in V_{{\MakeLowercase {\mathbf {g}}}_D} \times Q$, find $(\varDelta {\MakeLowercase {\mathbf {u}}}, \varDelta p) \in V_{{\MakeLowercase {\mathbf {0}}}} \times Q$ such that

20 $$\begin{aligned} a_k(\varDelta {\MakeLowercase {\mathbf {u}}}, \varDelta {\MakeLowercase {\mathbf {v}}}) + b_k(\varDelta p, \varDelta {\MakeLowercase {\mathbf {v}}})&= -R_\mathrm {upper}({\MakeLowercase {\mathbf {u}}}^k, p^k; \varDelta {\MakeLowercase {\mathbf {v}}}), \end{aligned}$$

21 $$\begin{aligned} b_k(\varDelta q, \varDelta {\MakeLowercase {\mathbf {u}}}) - c(\varDelta p, \varDelta q)&= -R_\mathrm {lower}({\MakeLowercase {\mathbf {u}}}^k, p^k;\varDelta q), \end{aligned}$$

where

$$\begin{aligned} a_k(\varDelta {\MakeLowercase {\mathbf {u}}}, \varDelta {\MakeLowercase {\mathbf {v}}})&:= \int \limits _{\varOmega _0} {\text {Grad}}\,\varDelta {\MakeLowercase {\mathbf {v}}} \varvec{S}_{\mathrm {tot},k} : {\text {Grad}}\,\varDelta {\MakeLowercase {\mathbf {u}}}\,\mathrm {d}\varvec{\mathbf {X}}\\&\quad +\, \int \limits _{\varOmega _0}\varvec{\varSigma }({\MakeLowercase {\mathbf {u}}}_k, \varDelta {\MakeLowercase {\mathbf {v}}}) : \mathbb {C}_{\mathrm {tot},k} : \varvec{\varSigma }({\MakeLowercase {\mathbf {u}}}_k, \varDelta {\MakeLowercase {\mathbf {u}}})\,\mathrm {d}\varvec{\mathbf {X}},\\ b_k(\varDelta p, \varDelta {\MakeLowercase {\mathbf {v}}})&:= \int \limits _{\varOmega _0} \varDelta p \pi (J_k) \varvec{F}^{-\top }_k : {\text {Grad}}\,\varDelta {\MakeLowercase {\mathbf {v}}} \,\mathrm {d}\varvec{\mathbf {X}}, \end{aligned}$$

with abbreviations

22 $$\begin{aligned} \varvec{F}_k&:= \varvec{F}({\MakeLowercase {\mathbf {u}}}_k),\nonumber \\ J_k&:= \det (\varvec{F}_k),\nonumber \\ \varvec{S}_{\mathrm {tot},k}&:= \left. \varvec{S}_{\mathrm {isc}}\right| _{{\MakeLowercase {\mathbf {u}}} ={\MakeLowercase {\mathbf {u}}}_k} + p_k \left. \varvec{S}_{\mathrm {vol}}\right| _{{\MakeLowercase {\mathbf {u}}} ={\MakeLowercase {\mathbf {u}}}_k}, \end{aligned}$$

23 $$\begin{aligned} \mathbb {C}_{\mathrm {tot},k}&:= \left. \mathbb {C}_{\mathrm {isc}}\right| _{{\MakeLowercase {\mathbf {u}}} ={\MakeLowercase {\mathbf {u}}}_k} + p_k \left. \mathbb {C}_{\mathrm {vol}}\right| _{{\MakeLowercase {\mathbf {u}}} = {\MakeLowercase {\mathbf {u}}}_k}, \nonumber \\ \mathbb {C}_\mathrm {vol}&:= k(J) \varvec{C}^{-1} \otimes \varvec{C}^{-1} - 2 \pi (J) \varvec{C}^{-1} \odot \varvec{C}^{-1},\nonumber \\ k(J)&:= J^2 \varTheta ''(J) + J \varTheta '(J), \end{aligned}$$

where $\mathbb {C}_{\mathrm {isc}}$ is given in (57). The derivation of the consistent linearization is lengthy but standard, we refer to [45, Chapter 8] for details. The definition of the higher order tensor and other abbreviations are given in “Appendix”.

### Review on solvability of the linearized problem

Since (20) and (21) is a linear saddle-point problem for each given $({\MakeLowercase {\mathbf {u}}}^k, p^k)$ we can rely on the well-established Babuška–Brezzi theory, see [15, 36, 67, 70]. The crucial properties to guarantee that the problem (20) and (21) is well-posed are continuity of all involved bilinear forms and the following three conditions:

(i) The *inf–sup condition*: there exists $c_1 > 0$ such that
  24 $$\begin{aligned} \underset{q \in Q}{\inf } \ \underset{{\MakeLowercase {\mathbf {v}}} \in V_{{\MakeLowercase {\mathbf {0}}}}}{\sup } \frac{b_k(q,{\MakeLowercase {\mathbf {v}}})}{{\left||{{\MakeLowercase {\mathbf {v}}}}\right||}_{V_{{\MakeLowercase {\mathbf {0}}}}} {\left||{q}\right||}_{Q}} \ge c_1. \end{aligned}$$
(ii) The *coercivity on the kernel condition*: there exists $c_2 > 0$ such that
  25 $$\begin{aligned} a_k({\MakeLowercase {\mathbf {v}}}, {\MakeLowercase {\mathbf {v}}}) \ge c_2 {\left||{{\MakeLowercase {\mathbf {v}}}}\right||}_{V_{{\MakeLowercase {\mathbf {0}}}}}^2&\text {for all } {\MakeLowercase {\mathbf {v}}} \in \ker B, \end{aligned}$$
  where
  $$\begin{aligned} \ker B := \left\{ {\MakeLowercase {\mathbf {v}}} \in V_{{\MakeLowercase {\mathbf {0}}}} : b_k(q, {\MakeLowercase {\mathbf {v}}}) = 0 ~\text {for all } q \in Q \right\} . \end{aligned}$$
(iii) *Positivity of*$c$: it holds
  26 $$\begin{aligned} c(q,q) \ge 0 \ \text {for all }q\in Q. \end{aligned}$$

Upon observing that $\varvec{F}^{-\top } : {\text {Grad}}\,{\MakeLowercase {\mathbf {v}}} = {\text {div}}\,{{\MakeLowercase {\mathbf {v}}}}$, see [45], we rewrite the bilinear form $b_k(q, {\MakeLowercase {\mathbf {v}}})$ as

27 $$\begin{aligned} b_k(q, {\MakeLowercase {\mathbf {v}}})&= \int \limits _{\varOmega _0} q \pi (J_k) \varvec{F}^{-\top }_k : {\text {Grad}}\,{\MakeLowercase {\mathbf {v}}} \,\mathrm {d}\varvec{\mathbf {X}}\nonumber \\&= \int \limits _{\varOmega _0} q \pi (J_k) {\text {div}}\,{{\MakeLowercase {\mathbf {v}}}}\,\mathrm {d}\varvec{\mathbf {X}}\nonumber \\&= \int \limits _{\varOmega _t} q \varTheta '(J_k) {\text {div}}\,{{\MakeLowercase {\mathbf {v}}}}\,\mathrm {d}{\MakeLowercase {\mathbf {x}}}. \end{aligned}$$

Assuming that $\varTheta '(J) \ge 1$, we can conclude the *inf–sup* condition from standard arguments, see [83, Section 5.2]. The positivity of the bilinear form *c* is always fulfilled. However, it is not possible to show the coercivity condition (25) for a general hyperelastic material or load configuration. Nevertheless, for some special cases it is possible to establish a result. We refer to [7, 8, 83] for a more detailed discussion. Henceforth, we will assume that our given input data is such that we stay in the range of stability of the problem. Examples for cases in which bilinear form $a_k$ lacks coercivity can be found in [83, Chapter 9] and [7, Section 4].

## Finite element approximation and stabilization

Let $\mathcal {T}_h$ be a finite element partitioning of $\overline{\varOmega }$ into subdomains, in our case either tetrahedral or convex hexahedral elements. The partitioning is assumed to fulfill standard regularity conditions, see [29]. Let $\hat{K}$ be the reference element, and for $K\in \mathcal {T}_h$ denote by $F_\mathrm {K}$ the affine, or trilinear mapping from $\hat{K}$ onto $K$. We assume that $F_\mathrm {K}$ is a bijection. For a tetrahedral element $K$ this can be assured whenever $K$ is non-degenerate, however, for hexahedral elements this may not necessary be the case, see [53] for details. Further, let $\hat{\mathbb {V}}$ and $\hat{\mathbb {Y}}$ denote two polynomial spaces defined over $\hat{K}$. We denote by

28 $$\begin{aligned} V_{h,0}&:= \left\{ {\MakeLowercase {\mathbf {v}}} \in H^1_0(\varOmega _0):{\MakeLowercase {\mathbf {v}}} = \hat{{\MakeLowercase {\mathbf {v}}}} \circ F_\mathrm {K}^{-1},\hat{{\MakeLowercase {\mathbf {v}}}} \in {[\hat{\mathbb {V}}]}^3,\forall K \in \mathcal {T}_h\right\} , \end{aligned}$$

29 $$\begin{aligned} Q_h&:= \left\{ q \in L^2(\varOmega _0): p = \hat{p}\circ F_\mathrm {K}^{-1}, \hat{p}\in \hat{\mathbb {Y}},\forall K\in \mathcal {T}_h\right\} , \end{aligned}$$

30 $$\begin{aligned} V_{h,{\MakeLowercase {\mathbf {g}}}_\mathrm {D}}&:= H^1_{{\MakeLowercase {\mathbf {g}}}_\mathrm {D}}(\varOmega _0) \cap V_{h,0}, \end{aligned}$$

the spaces needed for further analysis in the following sections.

### Nearly incompressible linear elasticity

See [16, 72, 73].

As a model problem we study the well-known equations for nearly incompressible linear elasticity. In this case it is assumed that $\varOmega :=\varOmega _0\approx \varOmega _t$. Then, the linear elasticity problem reads: find $({\MakeLowercase {\mathbf {u}}},p) \in V_{{\MakeLowercase {\mathbf {g}}}_D} \times Q$ such that

31 $$\begin{aligned} 2\mu \int \limits _{\varOmega } \varvec{\varepsilon }({\MakeLowercase {\mathbf {u}}}) : \varvec{\varepsilon }({\MakeLowercase {\mathbf {v}}}) \,\mathrm {d}{\MakeLowercase {\mathbf {x}}}+ \int \limits _{\varOmega }p {\text {div}}\,{\MakeLowercase {\mathbf {v}}} \,\mathrm {d}\varvec{\mathbf {x}}&= \int \limits _{\varOmega } {\MakeLowercase {\mathbf {f}}} \cdot {\MakeLowercase {\mathbf {v}}} \,\mathrm {d}\varvec{\mathbf {x}} \end{aligned}$$

32 $$\begin{aligned} \int \limits _{\varOmega } {\text {div}}\,{\MakeLowercase {\mathbf {u}}} q \,\mathrm {d}\varvec{\mathbf {x}} - \frac{1}{\lambda } \int \limits _{\varOmega } p q \,\mathrm {d}\varvec{\mathbf {x}}&= 0 \end{aligned}$$

for all $({\MakeLowercase {\mathbf {v}}}, q) \in V_{{\MakeLowercase {\mathbf {0}}}} \times Q$. Here, $\mu >0$ and $\lambda$ denote the Lamé parameters, and $\varvec{\varepsilon }({\MakeLowercase {\mathbf {v}}}) := \mathrm {sym}({\text {grad}}\,{\MakeLowercase {\mathbf {v}}})$.

The regularity of (31) and (32) is a classical result [75] and follows with the same arguments as for the Stokes equations. The discretized analogue of (31) and (32) is: find $({\MakeLowercase {\mathbf {u}}}_h, p_h) \in V_{h,{\MakeLowercase {\mathbf {g}}}_D} \times Q_h$ such that

33 $$\begin{aligned} 2\mu \int \limits _{\varOmega } \varvec{\varepsilon }({\MakeLowercase {\mathbf {u}}}_h) : \varvec{\varepsilon }({\MakeLowercase {\mathbf {v}}}_h) \,\mathrm {d}{\MakeLowercase {\mathbf {x}}}+ \int \limits _{\varOmega }p_h {\text {div}}\,{\MakeLowercase {\mathbf {v}}}_h \,\mathrm {d}\varvec{\mathbf {x}}&= \int _{\varOmega } {\MakeLowercase {\mathbf {f}}} \cdot {\MakeLowercase {\mathbf {v}}}_h \,\mathrm {d}\varvec{\mathbf {x}} \end{aligned}$$

34 $$\begin{aligned} \int \limits _{\varOmega } {\text {div}}\,{\MakeLowercase {\mathbf {u}}}_h q_h \,\mathrm {d}\varvec{\mathbf {x}} - \frac{1}{\lambda } \int \limits _{\varOmega } p_h q_h \,\mathrm {d}\varvec{\mathbf {x}}&= 0 \end{aligned}$$

for all $({\MakeLowercase {\mathbf {v}}}_h, q_h) \in V_{h,{\MakeLowercase {\mathbf {0}}}} \times Q_h$. Coercivity on the kernel condition (25) is a standard result for the case of nearly incompressible linear elasticity posed in the form (31)–(32) and (33)–(34). In the nonlinear case this is not true in general and will be addressed in Sect. 3.4. The crucial point for checking well-posedness of the discrete Eqs. (33) and (34) is the fulfillment of the *discrete inf–sup condition*, reading

35 $$\begin{aligned} \underset{q_h \in Q_h}{\inf }\underset{{\MakeLowercase {\mathbf {v}}}_h \in V_{h,{\MakeLowercase {\mathbf {0}}}}}{\sup } \frac{\int \limits _{\varOmega } q_h {\text {div}}\,{\MakeLowercase {\mathbf {v}}}_h\,\mathrm {d}{\MakeLowercase {\mathbf {x}}}}{{\left||{{\MakeLowercase {\mathbf {v}}}_h}\right||}_{V_{{\MakeLowercase {\mathbf {0}}}}} {\left||{q_h}\right||}_{Q}} > 0. \end{aligned}$$

The discrete *inf–sup* condition puts constraints on the choice of the spaces $V_{h,0}$ and $Q_h$. A finite element pairing fulfilling (35) is called a *stable pair*. A classic example for tetrahedral meshes would be the Taylor–Hood element. In this paper, we will focus on two different finite element pairings, the MINI element and a stabilized equal order element. The stabilized equal order pairing has been used in this context for pure tetrahedral meshes, see [22, 65]. To the best of the authors knowledge those elements have not been used in the present context for general tesselations.

### The pressure-projection stabilized equal order pair

In the following, we present a stabilized lowest equal order finite element pairing, adapted to nonlinear elasticity from the pairing originally introduced by Dohrmann and Bochev [14, 33] for the Stokes equations.

We choose $\hat{\mathbb {V}}$ and $\hat{\mathbb {Y}}$ in (28) and (29) as the space of linear (or trilinear) functions over $\hat{K}$. This choice of spaces is a textbook example of an unstable element, however, following [33], we can introduce a stabilized formulation of (33) and (34) by: find $({\MakeLowercase {\mathbf {u}}}_h,p_h) \in V_{h,{\MakeLowercase {\mathbf {g}}}_D} \times Q_h$ such that

36 $$\begin{aligned}&\mu \int \limits _{\varOmega } \varvec{\varepsilon }({\MakeLowercase {\mathbf {u}}}_h) : \varvec{\varepsilon }({\MakeLowercase {\mathbf {v}}}_h) \,\mathrm {d}\varvec{\mathbf {x}} + \int \limits _{\varOmega }p_h {\text {div}}\,{\MakeLowercase {\mathbf {v}}}_h \,\mathrm {d}\varvec{\mathbf {x}} = \int _{\varOmega } {\MakeLowercase {\mathbf {f}}} \cdot {\MakeLowercase {\mathbf {v}}}_h \,\mathrm {d}\varvec{\mathbf {x}}, \end{aligned}$$

37 $$\begin{aligned}&\int \limits _{\varOmega } {\text {div}}\,{\MakeLowercase {\mathbf {u}}}_h q_h \,\mathrm {d}\varvec{\mathbf {x}} - \frac{1}{\lambda } \int \limits _{\varOmega } p_h q_h \,\mathrm {d}\varvec{\mathbf {x}} \nonumber \\&\quad - \frac{1}{\mu ^*} s_h(p_h,q_h) = 0, \end{aligned}$$

for all $({\MakeLowercase {\mathbf {v}}}_h, q_h) \in V_{h,0} \times Q_h$, where

38 $$\begin{aligned} s_h(p_h,q_h) := \int \limits _{\varOmega }(p_h - \varPi _h p_h) (q_h - \varPi _h q_h)\,\mathrm {d}\varvec{\mathbf {x}} \end{aligned}$$

and $\mu ^*>0$ a suitable parameter. We note that the integral in (38) has to be understood as sum over integrals of elements of the tessellation. The projection operator $\varPi _h$ is defined element-wise for each $K \in \mathcal {T}_h$

$$\begin{aligned} \left. \varPi _h p_h\right| _{K} := \frac{1}{{|{K}|}_{}} \int \limits _{K}p_h\,\mathrm {d}\varvec{\mathbf {x}}. \end{aligned}$$

We can state the following results for this discrete problem:

#### Theorem 1

There exists a unique bounded solution to the discrete problem (36).

#### Theorem 2

Assume that ${\MakeLowercase {\mathbf {u}}} \in {[H^1_{{\MakeLowercase {\mathbf {g}}}_D}(\varOmega )]}^3 \cap {[H^2(\varOmega )]}^3$ and $p \in {\mathrm {L}^{2} (\varOmega )} \cap {\mathrm {H}^{1} (\varOmega )}$ solve the problem (31) and (32). Further, assume that $({\MakeLowercase {\mathbf {u}}}_h, p_h)$ are the solutions to the stabilized problem (36). Then there exists a constant $c_3$ independent of the mesh size $h$ and it holds:

39 $$\begin{aligned} {\left||{{\MakeLowercase {\mathbf {u}}} - {\MakeLowercase {\mathbf {u}}}_h}\right||}_{V} + {\left||{p-p_h}\right||}_{Q} \le c_3 h({\left\| {{\MakeLowercase {\mathbf {u}}}}\right\| _{{\mathrm {H}^{2} (\varOmega )}}}+{\left\| {p}\right\| _{{\mathrm {H}^{1} (\varOmega )}}}) \end{aligned}$$

#### Proof

Due to the similarity of the linear elasticity and the Stokes problem the proof follows from [14, Theorem 4.1, Theorem 5.1 and Corollary 5.2]. $\square$

### Discretization with MINI-elements

#### Tetrahedral elements

One of the earliest strategies in constructing a stable finite element pairing for discrete saddle-point problems arising from Stokes Equations is the MINI-Element, dating back to the works of Brezzi et al., see for example [3, 20]. In the case of Stokes the velocity ansatz space is enriched by suitable polynomial bubble functions. More precisely, if we denote by $\hat{\mathbb {P}}_1$ the space of polynomials with degree $\le 1$ over the reference tetrahedron $\hat{K}$, we will choose

$$\begin{aligned} \hat{\mathbb {V}}&= \hat{\mathbb {P}}_1 \oplus \{\hat{\psi }_\mathrm {B}\},\\ \hat{\mathbb {Y}}&= \hat{\mathbb {P}}_1, \\ \hat{\psi }_\mathrm {B}&:= 256 \xi _0 \xi _1 \xi _2 (1 - \xi _0 - \xi _1 - \xi _2), \end{aligned}$$

where $(\xi _0, \xi _1, \xi _2) \in \hat{K}$, see also [15]. Classical results [15] guarantee the stability of the MINI-Element for tetrahedral meshes. Due to compact support of the bubble functions, static condensation can be applied to remove the interior degrees of freedom during assembly. A short review on the static condensation process is given in “Appendix”. Hence, these degrees of freedom are not needed to be considered in the full global stiffness matrix assembly which is a key advantage of the MINI element.

#### Hexahedral meshes

In the literature mostly two dimensional quadrilateral tessellations, see for example [11, 15, 55], were considered for MINI element discretizations. In this case, the proof of stability relies on the so-called *macro-element technique* proposed by Stenberg [76].

To motivate our novel ansatz for hexahedral bubble functions, we will first give an overview of Stenbergs main results. A macro-element *M* is a connected set of elements in $\mathcal {T}_h$. Moreover, two macro-elements $M_1$ and $M_2$ are said to be equivalent if and only if they can be mapped continuously onto each other. Additionally, for a macro element *M* we define the spaces

40 $$\begin{aligned} \begin{aligned} \varvec{V}_{0,\mathrm {M}}&:= \left\{ {\MakeLowercase {\mathbf {v}}} \in {[H^1_0(M)]}^3 : {\MakeLowercase {\mathbf {v}}} = \hat{{\MakeLowercase {\mathbf {v}}}} \circ F_\mathrm {K}^{-1}, \right. \\&\left. \quad \hat{v} \in {[\hat{\mathbb {V}}]}^3,~K \subset M\right\} ,\\ P_\mathrm {M}&:= \left\{ p \in L^2(M):p = \hat{p}\circ F_\mathrm {K}^{-1},\hat{p} \in \hat{\mathbb {Y}},~K\subset M\right\} ,\\ N_\mathrm {M}&:= \left\{ p \in P_\mathrm {M} : \int \limits _{M} p {\text {div}}\,{\MakeLowercase {\mathbf {v}}}\,\mathrm {d}{\MakeLowercase {\mathbf {x}}}= 0, \forall {\MakeLowercase {\mathbf {v}}} \in \varvec{V}_{0,\mathrm {M}}\right\} . \end{aligned} \end{aligned}$$

Denote by $\varGamma _h$ the set of all edges in $\mathcal {T}_h$ interior to $\varOmega$. The macro-element partition $\mathcal {M}_h$ of $\varOmega$ then consists of a (not necessarily disjoint) partitioning into macro-elements ${\{M_i\}}_{i=1}^M$ with $\overline{\varOmega } = \bigcup _{i=1}^M \overline{M}_i$. The macro element technique is then described by the following theorem, see [76].

**Fig. 1:**
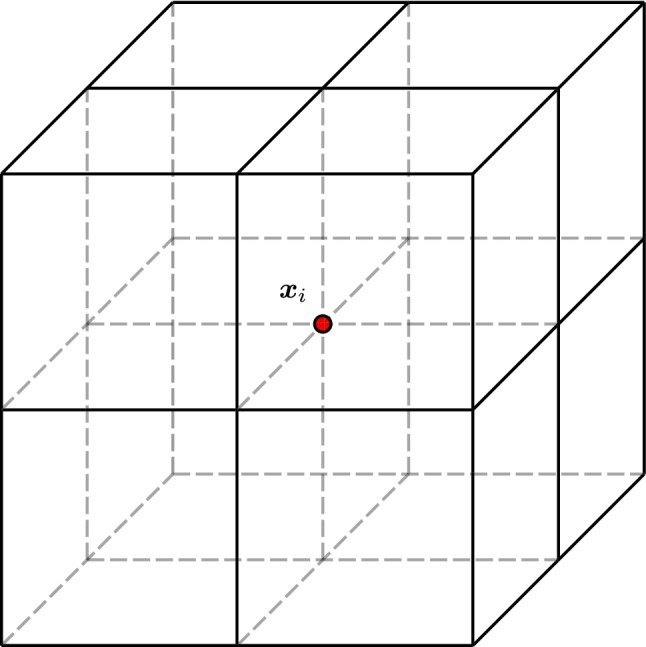
Macro-element definition for a mesh point ${\MakeLowercase {\mathbf {x}}}_i$

##### Theorem 3

Suppose that there is a fixed set of equivalence classes $\mathcal {E}_j$, $j=1,\ldots ,q$, of macro-elements, a positive integer *L*, and a macro-element partition $\mathcal {M}_h$ such that

1. for each $M_i \in \mathcal {E}_j$, $j=1,\ldots ,q$, the space $N_\mathrm {M}$ is one-dimensional consisting of functions that are constant on *M*;
2. each $M \in \mathcal {M}_h$ belongs to one of the classes $\mathcal {E}_i$, $i=1,2,\ldots ,q$;
3. each $K \in \mathcal {T}_h$ is contained in at least one and not more than *L* macro-elements of $\mathcal {M}_h$.
4. each $E \in \varGamma _h$ is contained in the interior of at least one and not more than *L* macro-elements of $\mathcal {M}_h$.

Then the discrete $\inf$–$\sup$-condition (35) holds.

**Fig. 2:**
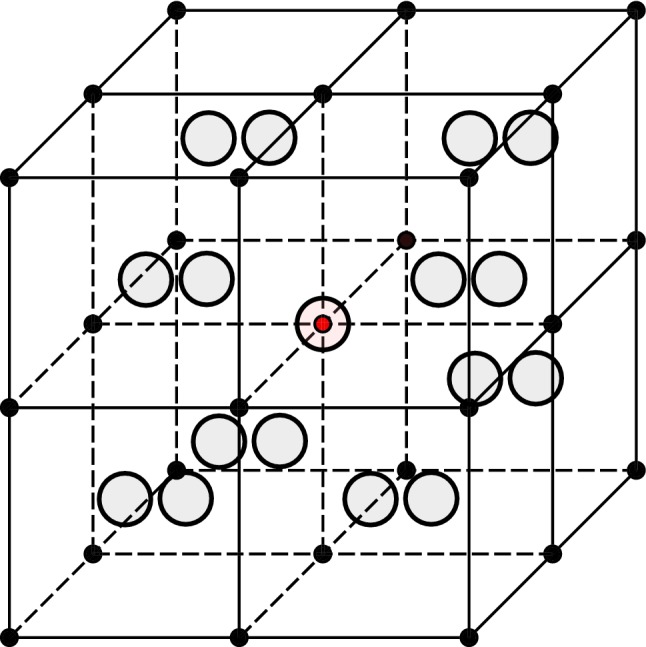
Macro-element distribution of degrees of freedom for ${\MakeLowercase {\mathbf {v}}}_h \in \varvec{V}_{0,\mathrm {M}}$ and $q_h \in P_\mathrm {M}$. Small filled dots correspond to $P_\mathrm {M}$ and bigger opaque circles correspond to $\varvec{V}_{0,\mathrm {M}}$

Conditions (M2)–(M4) are valid for a quasi-uniform tessellation of $\varOmega$ into hexahedral elements and, thus, it remains to show (M1). To this end, we consider a macro-element $M_i \in \mathcal {M}_h$ consisting of eight hexahedrons that share a common vertex ${\MakeLowercase {\mathbf {x}}}_i \in \varOmega$, see Fig. 1. A macro-element partitioning of this type fulfills conditions (M1)–(M3) from Theorem 3. We will next show, that Assumption (M1) depends on the choice of the bubble functions inside every $K \in M_i$. For ease of presentation and with no loss of generality we will assume that $M_i$ is a parallelepiped. This means that the mapping $F_{\mathrm {M}_i}$ from $\hat{K}$ onto $M_i$ is affine, so there exists an invertible matrix $\varvec{J}_i \in \mathbb {R}^{3\times 3}$ such that

$$\begin{aligned} {\MakeLowercase {\mathbf {x}}} = F_{\mathrm {M}_i}(\varvec{\xi }) = \varvec{J}_i \varvec{\xi }+ {\MakeLowercase {\mathbf {x}}}_0, \end{aligned}$$

where $\varvec{\xi }\in \hat{K} = {[-1,1]}^3$ and ${\MakeLowercase {\mathbf {x}}}_0$ is a given node of $M_i$. The case of $M_i$ not being the image of an affine mapping of $\hat{K}$ can be handled analogously, however, there are constraints on the invertibility of $F_{\mathrm {M}_i}$, see [53]. Let ${\{\psi _j\}}_{j=1}^8$ denote the standard trilinear basis functions on the unit hexahedron. These functions will serve as a basis for $P_{\mathrm {M}_i}$. For the space $\varvec{V}_{0,\mathrm {M}_i}$ we will chose one piecewise continuous trilinear ansatz function defined in ${\MakeLowercase {\mathbf {x}}}_i$ and for each sub-hexahedron we will add two bubble functions as degrees of freedom. The distribution of the degrees of freedom is depicted in Fig. 2. On $\hat{K}$ we will define the following two bubble functions

41 $$\begin{aligned} \hat{\phi }_\mathrm {B}^1&:= {(1-\xi _0)}^2{(1-\xi _1)}^2{(1-\xi _2)}^2 \hat{\psi }_{\alpha }, \end{aligned}$$

42 $$\begin{aligned} \hat{\phi }_\mathrm {B}^2&:= {(1-\xi _0)}^2{(1-\xi _1)}^2{(1-\xi _2)}^2 \hat{\psi }_{\beta }, \end{aligned}$$

where the indices $\alpha , \beta$ are chosen such that $\hat{\psi }_{\alpha }$ and $\hat{\psi }_{\beta }$ are two ansatz functions belonging to two diagonally opposite nodes. Having this, we will form a basis for $\varvec{V}_{0,\mathrm {M}_i}$ by gluing together the images of the basis functions of each sub-hexahedron. So we can write a basis for $\varvec{V}_{0,\mathrm {M}_i}$ as

43 $$\begin{aligned} V_{0,\mathrm {M}_i}&:= {\text {span}}\{\psi _{{\MakeLowercase {\mathbf {x}}}_i}, \phi _{\mathrm {B},1}^1, \phi _{\mathrm {B},2}^1,\ldots ,\phi _{\mathrm {B},1}^8, \phi _{\mathrm {B},2}^8\},\nonumber \\ \varvec{V}_{0,\mathrm {M}_i}&:= {[V_{0,\mathrm {M}_i}]}^3. \end{aligned}$$

Here, $\psi _{{\MakeLowercase {\mathbf {x}}}_i}$ corresponds to a piecewise trilinear ansatz function that has unit value in ${\MakeLowercase {\mathbf {x}}}_i$ and zero in all other nodes of $M_i$. Thus, we can calculate that $\dim (P_{\mathrm {M}_i}) = 27$ and $\dim (\varvec{V}_{0,\mathrm {M}_i}) = 51$. For ease of presentation we will rename the elements of (43) as ${\{\phi _i\}}_{i=1}^{17}$. Now, for $q_h \in P_{\mathrm {M}_i}$ and ${\MakeLowercase {\mathbf {v}}}_h \in \varvec{V}_{0,\mathrm {M}_i}$ we can write

$$\begin{aligned} \int \limits _{M_i} q_h {\text {div}}\,{\MakeLowercase {\mathbf {v}}}_h\,\mathrm {d}{\MakeLowercase {\mathbf {x}}}= \sum _{k=1}^{17}\sum _{l=1}^{27}\sum _{j=1}^3v_k^j q_l \int \limits _{M_i} {\text {grad}}\,_{{\MakeLowercase {\mathbf {x}}}} \phi _k[j] \psi _l\,\mathrm {d}{\MakeLowercase {\mathbf {x}}}. \end{aligned}$$

Next, we use the chain rule to get ${\text {grad}}\,_{{\MakeLowercase {\mathbf {x}}}} \phi _k = \varvec{J}_i^{-\top } \hat{{\text {grad}}\,}_{\varvec{\xi }}$ and a change of variables to obtain

$$\begin{aligned}&\sum _{k=1}^{17}\sum _{l=1}^{27}\sum _{j=1}^3 v_k^j q_l \int \limits _{M_i} {\text {grad}}\,_{{\MakeLowercase {\mathbf {x}}}} \phi _k[j] \psi _l\,\mathrm {d}{\MakeLowercase {\mathbf {x}}},\\&\quad =\sum _{k=1}^{17}\sum _{l=1}^{27}\sum _{j=1}^3v_k^j q_l \int \limits _{\hat{K}} \varvec{J}_i^{-\top }\hat{{\text {grad}}\,}_{\varvec{\xi }} \hat{\phi }_k[j] \hat{\psi }_j|\det {\varvec{J}_i}|\mathrm {d}\varvec{\xi }. \end{aligned}$$

This means we can find a matrix $\widetilde{\varvec{D}} \in \mathbb {R}^{27\times 51}$ such that

$$\begin{aligned} \int \limits _{M_i} q_h {\text {div}}\,{\MakeLowercase {\mathbf {v}}}_h\,\mathrm {d}{\MakeLowercase {\mathbf {x}}}= {\MakeLowercase {\mathbf {q}}}^\top \widetilde{\varvec{D}} {\MakeLowercase {\mathbf {v}}}, \end{aligned}$$

where ${\MakeLowercase {\mathbf {q}}}$ and ${\MakeLowercase {\mathbf {v}}}$ encode the nodal values of $q_h$ and ${\MakeLowercase {\mathbf {v}}}_h$. The following ordering will be employed for ${\MakeLowercase {\mathbf {v}}}$

$$\begin{aligned} {\MakeLowercase {\mathbf {v}}} = {\left( v_1^1,v_1^2,v_1^3,\ldots ,v_{17}^1,v_{17}^2,v_{17}^3\right) }^\top . \end{aligned}$$

To proof (M1) we need to show that the rank of the matrix $\widetilde{\varvec{D}}$ is 26. Due to the invertibility of $\varvec{J}_i$ the rank of the matrix $\widetilde{\varvec{D}}$ will remain unchanged by replacing $M_i$ by $\hat{K}$. Thus, it suffices to compute the rank of the matrix $\varvec{D}$ whose $j^{\mathrm {th}}$ row is defined by

$$\begin{aligned}&\left( \int \limits _{\hat{K}} \partial _{\xi _1}\phi _1\psi _1\mathrm {d}\varvec{\xi },\int \limits _{\hat{K}} \partial _{\xi _2}\phi _1\psi _1\mathrm {d}\varvec{\xi },\int \limits _{\hat{K}} \partial _{\xi _3}\phi _1\psi _1\mathrm {d}\varvec{\xi },\right. \\&\qquad \qquad ,\ldots , \\&\quad \left. \int \limits _{\hat{K}} \partial _{\xi _1}\phi _{17}\psi _j\mathrm {d}\varvec{\xi },\int \limits _{\hat{K}} \partial _{\xi _2}\phi _{17}\psi _j\mathrm {d}\varvec{\xi },\int \limits _{\hat{K}} \partial _{\xi _3}\phi _{17}\psi _j\mathrm {d}\varvec{\xi }\right) . \end{aligned}$$

By this formula the matrix $\varvec{D}$ can be explicitly calculated, e.g., by using software packages like *Mathematica*$^{\mathrm {TM}}$ and further analyzed. We can conclude that the rank of $\varvec{D}$ is 26 and thus (M1) holds and we can apply Theorem 3. A *Mathematica*$^{\mathrm {TM}}$ notebook containing computations discussed in this section is available upon request.

##### Remark 1

Contrary to the two-dimensional case studied in [11, 55] it is not sufficient to enrich the standard isoparametric finite element space for hexahedrons with only one bubble function. In this case both the spaces $\varvec{V}_{0,\mathrm {M}_i}$ and $P_{\mathrm {M}_i}$ have a dimension of 27, however, matrix $\varvec{D}$ has only rank 24.

##### Remark 2

Although not mentioned explicity, the stability of the MINI element holds also for mixed discretizations.

### Changes and limitations in the nonlinear case

One of the main differences between the linear and nonlinear case stems from the definition of the pressure *p* as remarked in [16]. Consider, as an example, the strain energy function for a nearly incompressible neo-Hookean material where

$$\begin{aligned} \overline{\varPsi }(\overline{\varvec{C}}) := \frac{\mu }{2}\left( \mathrm {tr}(\overline{\varvec{C}})-3\right) , \end{aligned}$$

with $\mu >0$ a material parameter. Then, $\varvec{S}_{\mathrm {tot}}$ and $\mathbb {C}_{\mathrm {tot}}$, evaluated at $({\MakeLowercase {\mathbf {u}}}_k, p_k) = ({\MakeLowercase {\mathbf {0}}}, 0)$, are given by

$$\begin{aligned} \varvec{S}_{\mathrm {tot}} = \varvec{0},\quad \mathbb {C}_{\mathrm {tot}} = 2\mu \varvec{I} \odot \varvec{I} - \frac{2\mu }{3} \varvec{I} \otimes \varvec{I}, \end{aligned}$$

independent of the choice of $\varTheta (J)$. Assuming that $\varOmega :=\varOmega _0\approx \varOmega _t$ we obtain from Eqs. (20) and (21) the following linear system

44 $$\begin{aligned} 2\mu \int \limits _{\varOmega } \varvec{\varepsilon }_\mathrm {d}({\MakeLowercase {\mathbf {u}}}) : \varvec{\varepsilon }_\mathrm {d}({\MakeLowercase {\mathbf {v}}}) \,\mathrm {d}{\MakeLowercase {\mathbf {x}}}+ \int \limits _{\varOmega }p {\text {div}}\,{\MakeLowercase {\mathbf {v}}} \,\mathrm {d}\varvec{\mathbf {x}}&= \int \limits _{\varOmega } {\MakeLowercase {\mathbf {f}}} \cdot {\MakeLowercase {\mathbf {v}}} \,\mathrm {d}\varvec{\mathbf {x}}, \end{aligned}$$

45 $$\begin{aligned} \int \limits _{\varOmega } {\text {div}}\,{\MakeLowercase {\mathbf {u}}} q \,\mathrm {d}\varvec{\mathbf {x}} - \frac{1}{\kappa } \int \limits _{\varOmega } p q \,\mathrm {d}\varvec{\mathbf {x}}&= 0, \end{aligned}$$

where $\varvec{\varepsilon }_\mathrm {d}({\MakeLowercase {\mathbf {u}}}) := \varvec{\varepsilon }({\MakeLowercase {\mathbf {u}}}) - \frac{1}{3}\mathrm {div}({\MakeLowercase {\mathbf {u}}}) \varvec{I}$. While the pressure in formulation (31) and (32) is usually denoted as *Herrmann pressure* [44], above formulation (44) and (45) uses the so-called *hydrostatic pressure*.

The arguments to prove the *inf–suf* condition for this linear problem remains the same as for (31) and (32). For the extension of the *inf–suf* condition to the nonlinear case we already stated earlier in Eq. (27) that

$$\begin{aligned} b_k(q_h,{\MakeLowercase {\mathbf {v}}}_h) = \int \limits _{\varOmega _{t,h}} q_h \varTheta '(J_h) {\text {div}}\,{\MakeLowercase {\mathbf {v}}}_h\,\mathrm {d}{\MakeLowercase {\mathbf {x}}}. \end{aligned}$$

Here, $\varOmega _{t,h}$ is the approximation of the real current configuration $\varOmega _t$. Our conjecture is that stability of the chosen elements is given provided sufficient fine discretizations and volumetric functions $\varTheta (J)$ fulfilling $\varTheta '(J) \ge 1$. However, we can not offer a rigorous proof of this, and rely on our numerical results which showed no sign of numerical instabilities.

Concerning well-posedness of (44)–(45), it was noted in [16], that the coercivity on the kernel condition (25) does not hold in general, which makes the formulation with hydrostatic pressure not well-posed in general. However, it remains well-posed for strictly divergence-free finite elements or pure Dirichlet boundary conditions. This has also been observed by other authors, see [52, 81]. Even if the coercivity on the kernel condition can be shown for the hydrostatic, nearly incompressible linear elastic case this result may not transfer to the nonlinear case. Here, this condition is highly dependent on the chosen nonlinear material law and for the presented benchmark examples (Sect. 4) we did not observe any numerical instabilities.

For an in-depth discussion we refer the interested reader to [7, 8]. A detailed discussion on Herrmann-type pressure in the nonlinear case is presented in [72, 73].

To show well-posedness for the special case of the presented MINI element discretizations we rely on results given in [16, Section 4]. There it is shown, that discrete coercivity on the kernel holds, provided that a rigid body mode is the only function that renders

$$\begin{aligned} a({\MakeLowercase {\mathbf {u}}}_h, {\MakeLowercase {\mathbf {v}}}_h) := \int \limits _{\varOmega _h} \varvec{\varepsilon }_d({\MakeLowercase {\mathbf {u}}}_h) : \varvec{\varepsilon }_d({\MakeLowercase {\mathbf {v}}}_h)\,\mathrm {d}{\MakeLowercase {\mathbf {x}}}\end{aligned}$$

from (44) to (45) zero. We could obtain this result following the same procedure outlined in [16] for both hexahedral and tetrahedral MINI elements. A *Mathematica*$^{\mathrm {TM}}$ notebook containing the computations discussed is available upon request.

In the case of the pressure-projection stabilization we will modify Eq. (17) using the stabilization term (38)

$$\begin{aligned} R_\mathrm {lower}({\MakeLowercase {\mathbf {u}}}_h, p_h;\varDelta q_h)&:= b_{\mathrm {vol}}({\MakeLowercase {\mathbf {u}}}_h; \varDelta q_h) - c(p_h,\varDelta q_h) \\&\qquad -\, \frac{1}{\mu ^*} s_h(p_h,q_h). \end{aligned}$$

Here, the stabilization parameter $\mu ^*>0$ is supposed to be large enough and will be specifically defined for each nonlinear material considered. Note, that by modifying the definition of the lower residual, we introduced a mesh dependent perturbation of the original residual. An estimate of the consistency error caused by this is not readily available and will be the topic of future research. However, results and comparisons to benchmarks in Sect. 4 suggest that this error is negligible for the considered problems as long as $\mu ^*$ is well-chosen. If not specified otherwise we chose

- $\mu ^*=\mu$ for neo-Hookean materials and
- $\mu ^*=c_1$ for Mooney–Rivlin materials

in the results section. For the pressure-projection stabilized equal order pair we can not transfer the results from the linear elastic case to the non-linear case, as the proof of well-posedness relies on the coercivity of $a_k({\MakeLowercase {\mathbf {u}}}, {\MakeLowercase {\mathbf {v}}})$ which can not be concluded for this formulation. However, no convergence issues occured in the numerical examples given in Sect. 4.

The considerable advantage of the MINI element is that there are no modifications needed and that no additional stabilization parameters are introduced into the system.

### Changes and limitations in the transient case

The equations presented in Sect. 2 are not yet suitable for transient simulations. To include this feature we modify the nonlinear variational problem (18) in the following way:

46 $$\begin{aligned} R_\mathrm {upper}^\mathrm {trans}({\MakeLowercase {\mathbf {u}}},p; \varDelta {\MakeLowercase {\mathbf {v}}})&:= \rho _0 \int \limits _{\varOmega _0} \ddot{{\MakeLowercase {\mathbf {u}}}} \cdot \varDelta {\MakeLowercase {\mathbf {v}}}\,\mathrm {d}{\MakeLowercase {\mathbf {x}}}+ R_\mathrm {upper}({\MakeLowercase {\mathbf {u}}}, p; \varDelta v), \end{aligned}$$

47 $$\begin{aligned} R_\mathrm {lower}^\mathrm {trans}({\MakeLowercase {\mathbf {u}}},p; \varDelta q)&:= R_\mathrm {lower}({\MakeLowercase {\mathbf {u}}}, p; \varDelta q). \end{aligned}$$

For time discretization we considered a generalized-$\alpha$ method, see [28] and also the “Appendix” for a short summary. Due to the selected formulation, the resulting ODE system turns out to be of degenerate hyperbolic type. Hence, we implemented a variant of the generalized-$\alpha$ method as proposed in [50] and using that we did not observe any numerical issues in our simulations. Note, that other groups have proposed a different treatment of the incompressibility constraints in the case of transient problems, see [66, 71] for details.

## Numerical examples

While benchmark cases presented in this section are fairly simple, mechanical applications often require highly resolved meshes. Thus, efficient and massively parallel solution algorithms for the linearized system of equations become an important factor to deal with the resulting computational load. After discretization, at each Newton–Raphson step a block system of the form

$$\begin{aligned} \begin{pmatrix} \varvec{K}_h &{}\quad \varvec{B}_h^\top \\ \varvec{B}_h &{}\quad \varvec{C}_h \end{pmatrix} \begin{pmatrix} \varDelta {\MakeLowercase {\mathbf {u}}} \\ \varDelta {\MakeLowercase {\mathbf {p}}} \end{pmatrix} = \begin{pmatrix} -\varvec{\mathbf {R}}_\mathrm {upper} \\ -\varvec{\mathbf {R}}_\mathrm {lower} \end{pmatrix} \end{aligned}$$

has to be solved. In that regard, we used a generalized minimal residual method (GMRES) and efficient preconditioning based on the PCFIELDSPLIT^1^ package from the library *PETSc* [12] and the incorporated solver suite *hypre/BoomerAMG* [43]. By extending our previous work [5] we implemented the methods in the finite element code *Cardiac Arrhythmia Research Package* (CARP) [82].

**Fig. 3:**
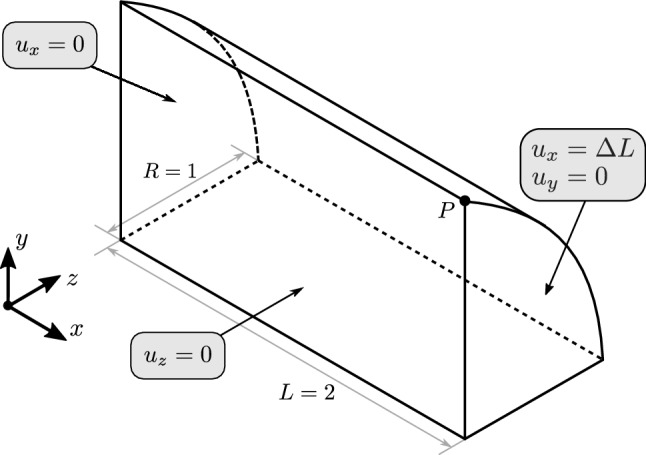
*Analytic solution:* geometry and boundary conditions

**Fig. 4:**
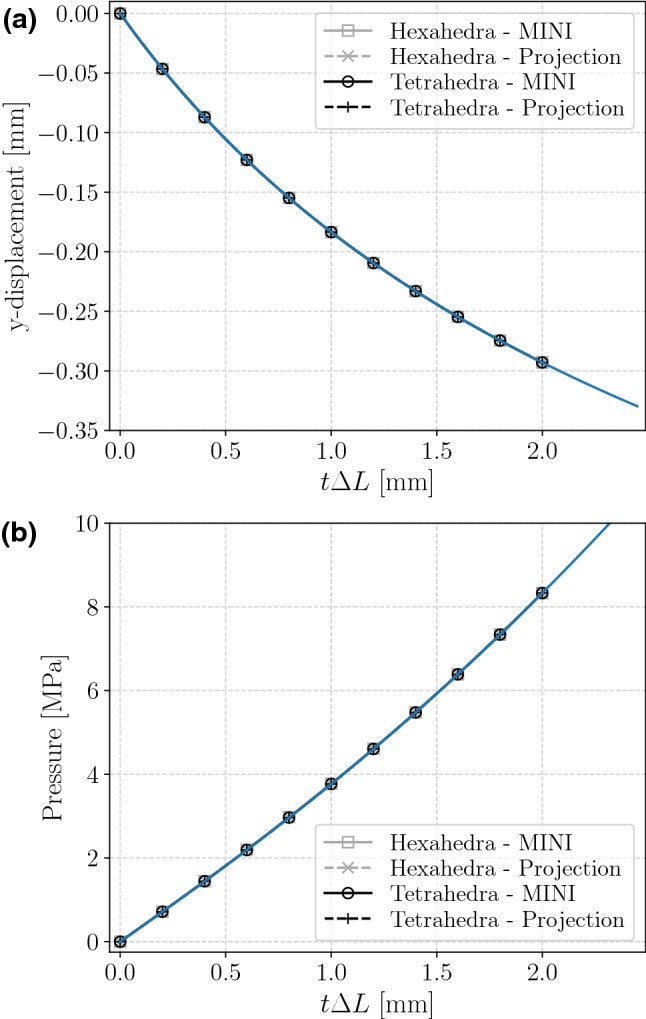
*Analytic solution:***a***y*-component of displacement and **b** pressure at point $P={(2,0,1)}^\top$. Simulation results of all proposed formulations are in perfect alignment with the analytic solution printed in blue. (Color figure online)

### Analytic solution

To verify our implementation we consider a very simple uniaxial tension test, see also [83, Sec. 10.1]. The computational domain is described by one eighth part of a cylinder with length $L=2\,\hbox {mm}$, and radius $R=1\,\hbox {mm}$

$$\begin{aligned} \varOmega _\mathrm {cyl,0} := \left\{ {\MakeLowercase {\mathbf {x}}} \in [0,L] \times {[0,R]}^2 : y^2+z^2\le R\right\} , \end{aligned}$$

see Fig. 3. This cylinder is stretched to a length of $L+\varDelta L$, with $\varDelta L = 2\,\hbox {mm}$.

**Fig. 5:**
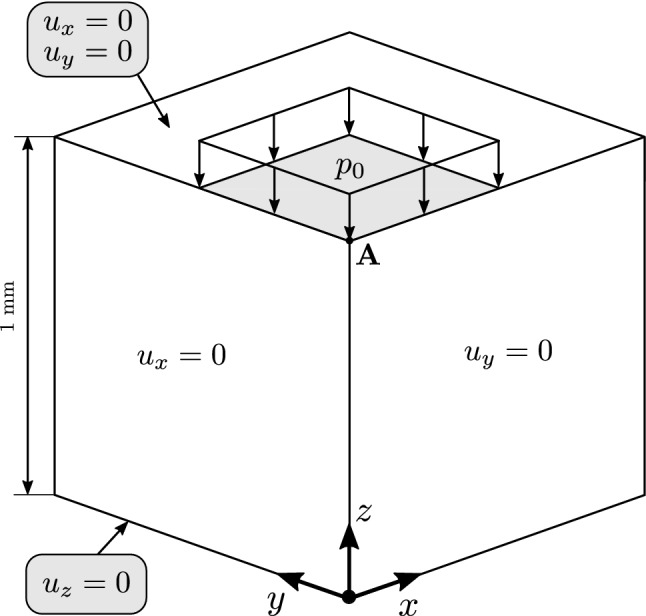
*Block under compression:* geometry and boundary conditions

**Table 1:** Properties of *cube meshes* used in Sect. 4.2

Hexahedral meshes			Tetrahedral meshes		
$\ell$	Elements	Nodes	$\ell$	Elements	Nodes
1	512	729	1	3072	729
2	4096	4913	2	24,576	4913
3	32,768	35,937	3	196,608	35,937
4	262,144	274,625	4	1,572,864	274,625
5	2,097,152	2,146,689	5	12,582,912	2,146,689

**Fig. 6:**
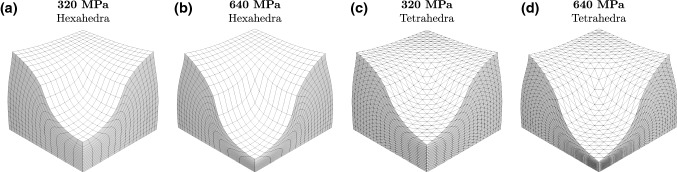
*Block under compression:* deformed meshes of hexahedral (**a**, **b**) and tetrahedral (**c**, **d**) elements for the $\ell =2$ mesh in Table 1 at load level $p=320\,\hbox {MPa}$ (**a**, **c**) and load level $p=640\,\hbox {MPa}$ (**b**, **d**)

**Fig. 7:**
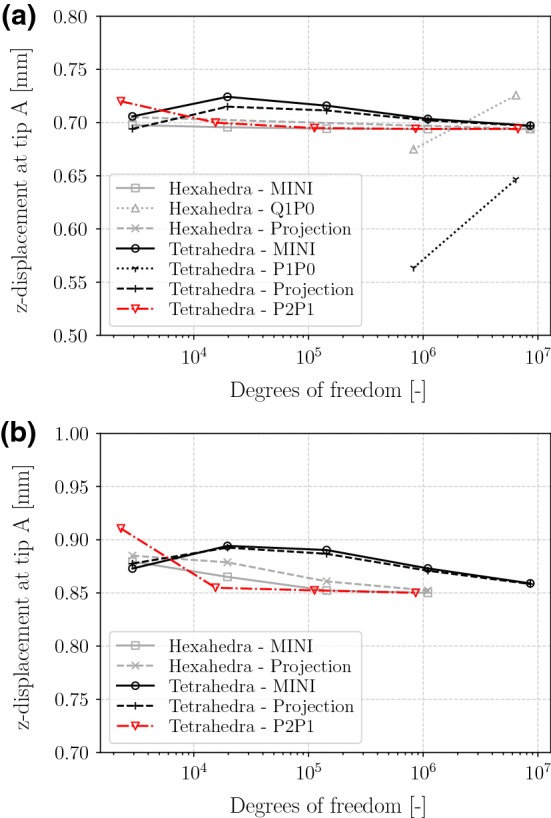
*Block under compression:* vertical displacement at point **A** versus number of degrees of freedom in a logarithmic scale at load level **a**$p_0=320\,\hbox {MPa}$ and **b**$p_0=640\,\hbox {MPa}$. Results for the MINI element and the pressure-projection stabilization are compared to classical choices of elements, i.e., $\mathbb {Q}_1 - \mathbb {P}_0$ hexahedral elements, $\mathbb {P}_1 - \mathbb {P}_0$ tetrahedral elements, and Taylor–Hood ($\mathbb {P}_2 - \mathbb {P}_1$) tetrahedral elements. For case **b** the choice of $\mathbb {Q}_1 - \mathbb {P}_0$ and $\mathbb {P}_1 - \mathbb {P}_0$ elements did not give reasonable results and were thus omitted

We chose a neo-Hookean material

$$\begin{aligned} \varPsi (\varvec{C}) = \frac{\mu }{2}\left( \mathrm {tr}(\overline{\varvec{C}}) - 3\right) + \frac{\kappa }{2} {\ln (J)}^2, \end{aligned}$$

with $\mu =7.14\,\hbox {MPa}$ and impose full incompressiblity, i.e., $1/\kappa =0$. For this special case, an analytic solution can be computed by

$$\begin{aligned} {\MakeLowercase {\mathbf {u}}}&= (t x, \varDelta R(t) y, \varDelta R(t) z),\\ p(t)&= \frac{\mu }{3}\left( {\left( 1+\frac{t \varDelta L}{L}\right) }^2 -{\left( 1+\frac{t \varDelta L}{L}\right) }^{-1}\right) ,\\ \varDelta R(t)&= {\left( 1+\frac{t\varDelta L}{L}\right) }^{-\frac{1}{2}} - 1, \end{aligned}$$

where $t \in [0,1]$ corresponds to the load increment. Two meshes consisting of 5420 points and 4617 hexahedral or 27,702 tetrahedral elements were used. We performed 20 incremental load steps with respect to $\varDelta L$. In Fig. 4 it is shown that the results of the numerical simulations render identical results for all the chosen setups and are in perfect agreement with the exact solution plotted in blue.

### Block under compression

The computational domain, studied by multiple authors, see, e.g., [23, 58, 63], consists of a cube loaded by an applied pressure in the center of the top face; see Fig. 5. A quarter of the cube is modeled, where symmetric Dirichlet boundary conditions are applied to the vertical faces and the top face is fixed in the horizontal plane.

**Table 2:** *Block under compression:* comparison of computational times for different discretizations. Timings were obtained using (a) 48 cores and (b) 192 cores on ARCHER, UK. Coarser grids, see Table 1, are used for Taylor–Hood elements $\mathbb {P}_2- \mathbb {P}_1$ to compare computational times for a similar number of degrees of freedom (DOF)

Discretization	Grid	DOF (Mio.)	Tet. (s)	Hex. (s)
(a)				
Projection	$\ell =4$	1.098	330	438
MINI	$\ell =4$	1.098	873	655
$\mathbb {P}_2- \mathbb {P}_1$	$\ell =3$	0.860	1202	–
(b)				
Projection	$\ell =5$	8.587	2488	2192
MINI	$\ell =5$	8.587	3505	4640
$\mathbb {P}_2 - \mathbb {P}_1$	$\ell =4$	6.715	27,154	–

**Fig. 8:**
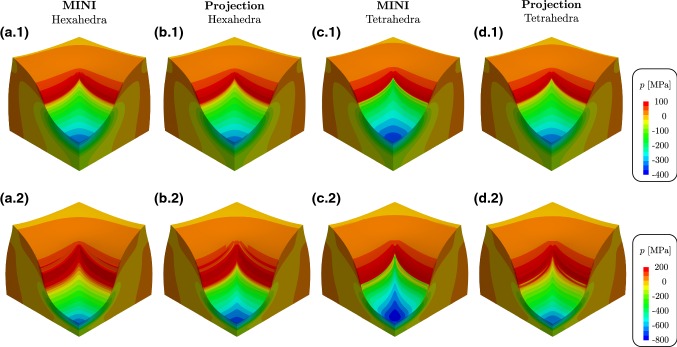
*Block under compression:* comparison of hexahedral (**a**, **b**) and tetrahedral (**c**, **d**) elements with bubble-based (**a**, **c**) and projection-based (**b**, **d**) stabilization. Shown is the pressure contour on the deformed mesh at load level $p=320\,\hbox {MPa}$ in the first row and $p=640\,\hbox {MPa}$ in the second row

The same neo-Hookean material model as in [58] is used:

$$\begin{aligned} \varPsi (\varvec{C})&=\frac{1}{2}\mu \left( {\text {tr}}(\varvec{C})-3\right) -\mu \ln J + \frac{\lambda }{2}{(\ln J)}^2, \end{aligned}$$

with material parameters $\lambda =400{,}889.806\,\hbox {MPa}$, $\mu =80.194\,\hbox {MPa}$. To test mesh convergence the simulations were computed on a series of uniformly refined tetrahedral and hexahedral meshes, see Table 1. Figure 6 shows the deformed meshes for the level $\ell =2$ with loads $p_0=320\,\hbox {MPa}$ and $p_0=640\,\hbox {MPa}$, respectively. In all cases discussed in this section we used 10 loading steps to arrive at the target pressure $p_0$. As a measure of the compression level the vertical displacement of the node at the center of the top surface, i.e. the edge point **A** of the quarter of the cube, is plotted in Fig. 7. Small discrepancies can be attributed to differences in the meshes for tetrahedral and hexahedral grids, however, the different stabilization techniques yield almost the same results for finer grids. Note, that the displacements at the edge point **A** obtained using the simple $\mathbb {Q}_1 - \mathbb {P}_0$ hexahedral and $\mathbb {P}_1 - \mathbb {P}_0$ tetrahedral elements seem to be in a similar range compared to the other approaches. The overall displacement field, however, was totally inaccurate rendering $\mathbb {Q}_1 - \mathbb {P}_0$ and $\mathbb {P}_1 - \mathbb {P}_0$ elements an inadequate choice for this benchmark problem. The solution for Taylor–Hood ($\mathbb {P}_2 - \mathbb {P}_1$) tetrahedral elements was obtained using the FEniCS project [2]. Here, as a linear solver, we used a GMRES solver with preconditioning similar to the MINI and projection-based approach, see first paragraph of Sect. 4. The PCFIELDSPLIT and *hypre/BoomerAMG* settings were slightly adapted to optimize computational performance for quadratic ansatz functions. We comparing simulations with about the same number of degrees of freedom, not accuracy as, e.g., in [25]. For coarser grids computational times were in the same time range for all approaches; see, e.g., the cases with approximately $10^6$ degrees of freedom and target pressure of $p_0=320\,\hbox {mmHg}$ in Table 2(a). For the simulations with the finest grids with approximately $10^7$ degrees of freedom, however, we could not find a setting for the Taylor–Hood elements that was competitive to MINI and pressure-projection stabilizations. The computational times to arrive at the target pressure of $p_0=320\,\hbox {mmHg}$ using 192 cores on ARCHER, UK were about 10 times higher for Taylor–Hood elements using FEniCS, see Table 2(b). We attribute that to a higher communication load and higher memory requirements of the Taylor–Hood elements: memory to store the block stiffness matrices was approximately 2.5 times higher for Taylor–Hood elements compared to MINI and projection-stabilization approaches (measured using the MatGetInfo^2^ function provided by PETSc). Note, that although we used the same linear solvers, the time comparisons are not totally just as results were obtained using two different finite element solvers, CARP and FEniCS. Note also, that timings are usually very problem dependent and for this block under compression benchmark high accuracy was already achieved with coarse grids for hexahedral and Taylor–Hood discretizations.

**Fig. 9:**
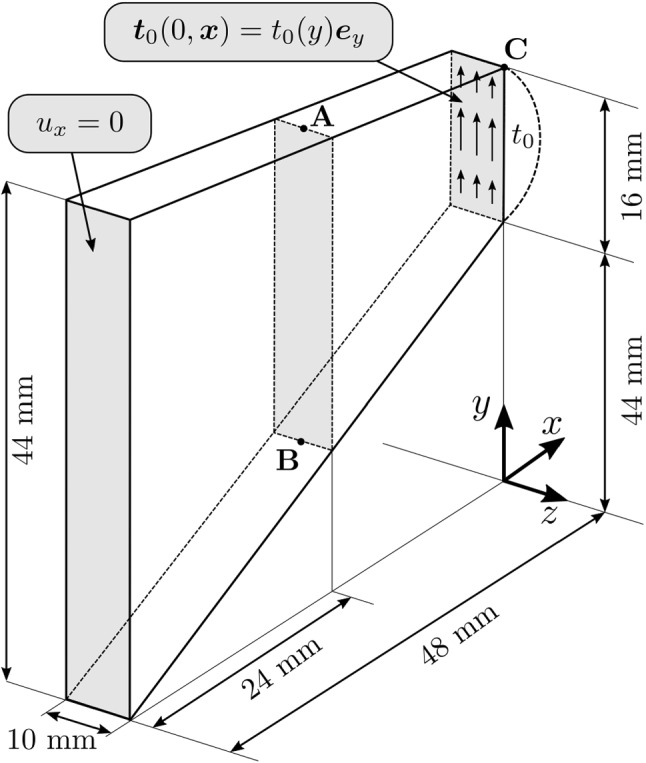
*Cook-type cantilever problem:* geometry and boundary conditions

**Fig. 10:**
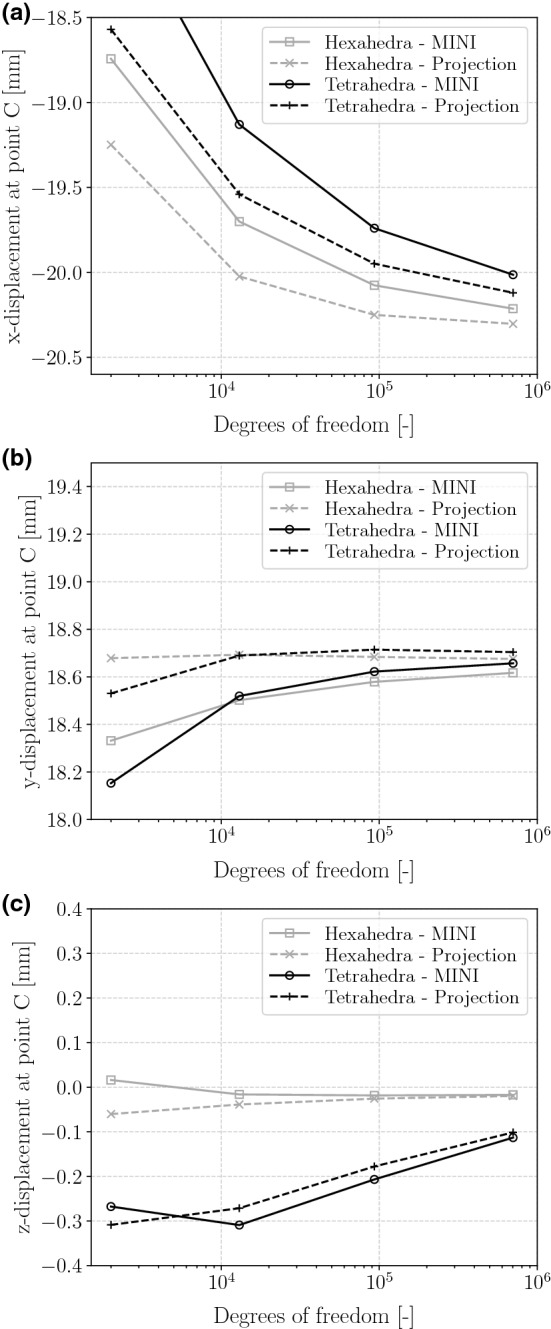
*Cook-type cantilever problem:* displacements $u_x$, $u_y$, and $u_z$ at point **C** versus the number of degrees of freedom in a logarithmic scale using the fully incompressible formulation

For a further analysis regarding computational costs of the MINI element and the pressure-projection stabilization, see Sect. 4.4.

In Fig. 8 the hydrostatic pressure is plotted for the MINI element and the projection-based stabilization. These results are very smooth in all cases and agree well with those published in [23, 35, 58, 63].

**Table 3:** Properties of *cantilever meshes* used in Sect. 4.3

Hexahedral meshes			Tetrahedral meshes		
$\ell$	Elements	Nodes	$\ell$	Elements	Nodes
1	324	500	1	1944	500
2	2592	3249	2	15,552	3249
3	20,736	23,273	3	124,416	23,273
4	165,888	175,857	4	995,328	175,857

### Cook-type cantilever problem

In this section, we analyze the same Cook-type cantilever beam problem presented in [17, 69], see also Fig. 9. Displacements at the plane $x=0\,\hbox {mm}$ are fixed. At the plane $x=48\,\hbox {mm}$ a parabolic load, which takes its maximum at $t_0=300\,\hbox {kPa}$, is applied. Note, that this in-plane shear force in y-direction is considered as a dead load in the deformation process. To compare to results in [69] the same isotropic strain energy function was chosen

$$\begin{aligned} \varPsi ^\mathrm {iso}(\varvec{C})=c_{1}{({\text {tr}}\varvec{C})}^2 + c_{2}{( {({\text {tr}}\varvec{C})}^2 - {\text {tr}}(\varvec{C}^2))}^2 - \gamma \ln (J), \end{aligned}$$

with material properties $c_1=21\,\hbox {kPa}$, $c_2=42\,\hbox {kPa}$, and $\gamma =12c_1+24c_2$ to satisfy the condition of a stress-free reference geometry.

**Fig. 11:**
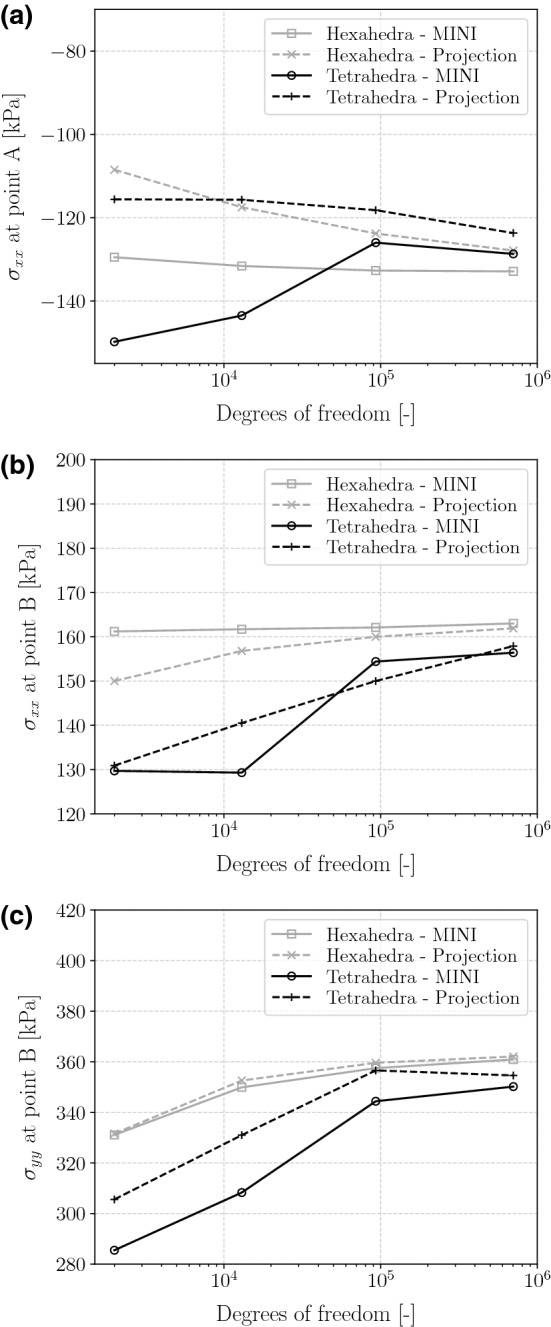
*Cook-type cantilever problem:* stresses $\sigma _{xx}$ at (left) point **A** and (middle) point **B** and $\sigma _{yy}$ at (right) point **B** versus the number of degrees of freedom in a logarithmic scale using the fully incompressible formulation

**Fig. 12:**
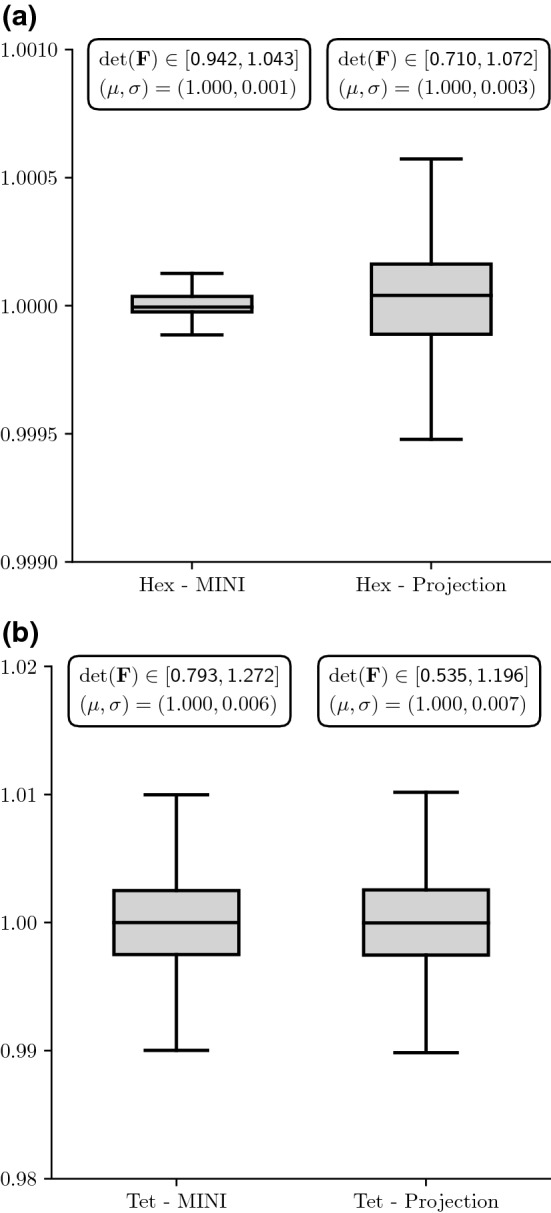
*Cook-type cantilever problem:* boxplots showing the distribution of $J=\det (\varvec{F})$ for **a** hexahedral and **b** tetrahedral elements. Additionally, the minimal and maximal value, as well as the mean ($\mu$) and the standard deviation ($\sigma$) is given for each setting

We chose a fully incompressible material, hence,

$$\begin{aligned} \varPsi ^\mathrm {vol}(\varvec{C})=\frac{\kappa }{2}{\left( J-1\right) }^2, \end{aligned}$$

with $1/\kappa =0$. First, mesh convergence with respect to resulting displacements is analyzed for the tetrahedral and hexahedral meshes with discretization details given in Table 3.

Displacements $u_x$, $u_y$, and $u_z$ at point $\mathbf {C}$ are shown in Fig. 10. The proposed stabilization techniques give comparable displacements in all three directions and also match results published in [17, 69]. Mesh convergence can also be observed for the stresses $\sigma _{xx}$ at point $\mathbf {A}$ and $\mathbf {B}$ and $\sigma _{yy}$ at point $\mathbf {B}$, see Fig. 11. Again, results match well those presented in [17, 69]. Small discrepancies can be attributed to the fully incompressible formulation used in our work and differences in grid construction.

**Fig. 13:**
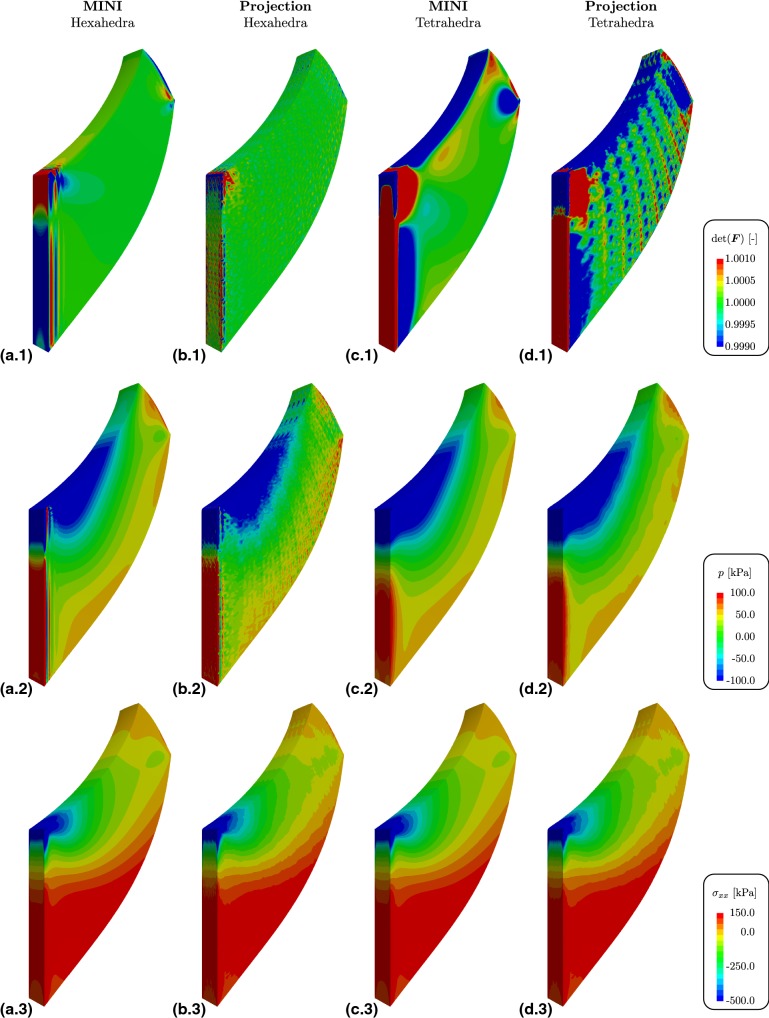
*Cook-type cantilever problem:* comparison of hexahedral (**a**, **c**) and tetrahedral (**b**, **d**) elements with bubble-based (**a**, **b**) and projection-based (**c**, **d**) stabilization. Shown is the distribution of $J=\det (\varvec{F})$ (first row); distribution of the hydrostatic pressure *p* (second row) in kPa; and the distribution of the stress $\sigma _{xx}$ (third row) in kPa for the fully incompressible formulation

In Figs. 12 and 13a the distribution of $J=\det (\varvec{F})$ is shown to provide an estimate of how accurately the incompressibility constraint is fulfilled by the proposed stabilization techniques. For most parts of the computational domains the values of *J* are close to 1, however, hexahedral meshes and here in particular the MINI element maintain the condition of $J \approx 1$ more accurately on the element level. Note, that for all discretizations the overall volume of the cantilever remained unchanged at $14{,}400\,\hbox {mm}^3$, rendering the material fully incompressible on the domain level.

Figure 13 gives a comparison of several computed values in the deformed configuration of Cook’s cantilever for the finest grids ($\ell =4$). Slight pressure oscillations in Fig. 13b on the domain boundary for the MINI element are to be expected, see [74]; this also affects the distribution of *J* in Fig. 13a. A similar checkerboard pattern is present for the projection based stabilization.

In the third row of Fig. 13 we compare the stresses $\sigma _{xx}$ for the different stabilization techniques. We can observe slight oscillations for the the projection-based approach, whereas the MINI element gives a smoother solution. Compared to results in [69, Figure 10] the $\sigma _{xx}$ stresses have a similar contour but are slightly higher. As before, we attribute that to the fully incompressible formulation in our paper compared to the quasi-incompressible formulation in [69].

### Twisting column test

**Fig. 14:**
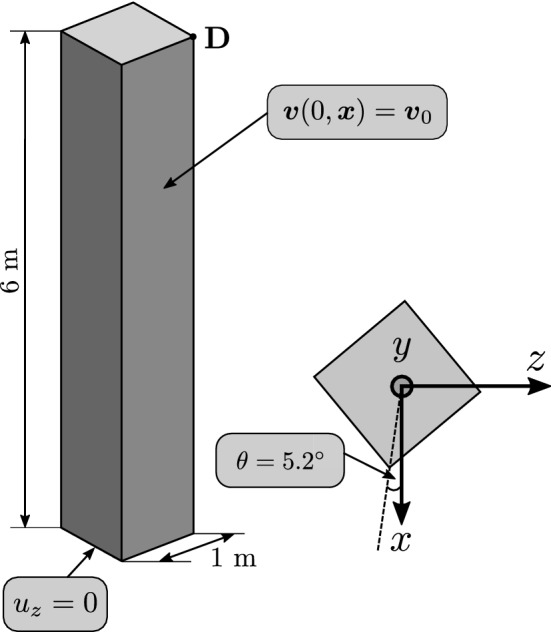
*Twisting column test:* geometry and boundary conditions

Finally, we show the applicability of our stabilization techniques for the transient problem of a twisting column [1, 40, 71]. The initial configuration of the geometry is depicted in Fig. 14. There is no load prescribed and the column is restrained against motion at its base. A twisting motion is applied to the domain by means of the following initial condition on the velocity

$$\begin{aligned} {\MakeLowercase {\mathbf {v}}}({\MakeLowercase {\mathbf {x}}}, 0) = {\MakeLowercase {\mathbf {v}}}(x,y,z, 0) = 100\sin \left( \frac{\pi y}{12}\right) {\left( z, 0, -x\right) }^\top \,\hbox {m/s}, \end{aligned}$$

for $y\in \left[ 0,6\right] \,\hbox {m}$. To avoid symmetries in the problem the column is rotated about the *z*-axes by an angle of $\theta =5.2{^{\circ }}$.

**Fig. 15:**
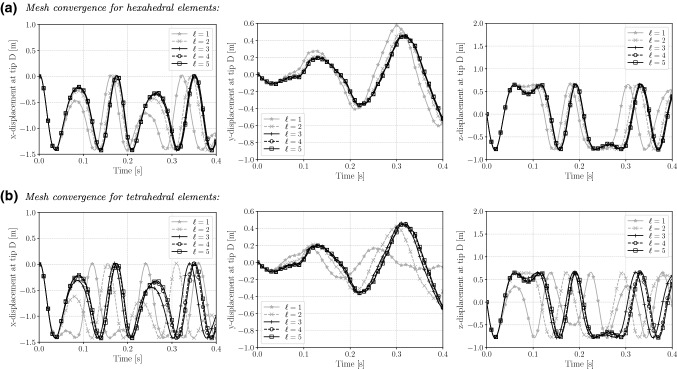
*Twisting column test:* mesh convergence for **a** hexahedral and **b** tetrahedral elements. Shown are displacements $u_x$, $u_y$, and $u_z$ at tip **D** versus time. For experiments depicted the incompressible formulation with MINI elements was chosen. At finer levels of refinement $\ell =3,4,5$ (in black) results converge to a solution for each displacement direction

**Fig. 16:**
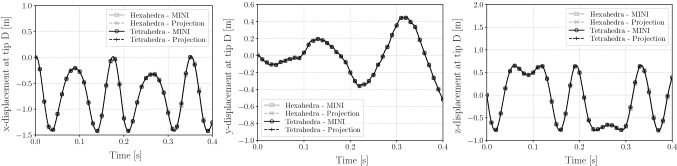
*Twisting column test:* comparison of stabilization techniques for the finest grids ($\ell =5$). Shown are displacements $u_x$, $u_y$, and $u_z$ at tip **D** versus time. Both MINI elements (dashed line) and projection-based stabilization (dashed lines) render almost identical results for hexahedral (in gray) and tetrahedral elements (in black)

**Fig. 17:**
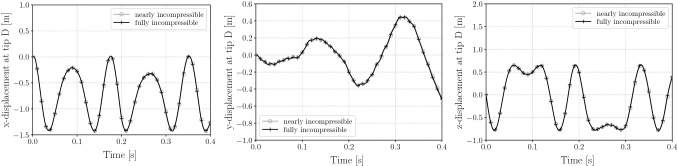
*Twisting column test:* comparison of nearly and fully incompressible formulation for the finest tetrahedral grids ($\ell =5$) and MINI elements. Displacements $u_x$, $u_y$, and $u_z$ are almost identical for the whole simulation duration of $0.4\,\hbox {s}$

We chose the neo-Hookean strain-energy

$$\begin{aligned} \varPsi (\varvec{C}) = \frac{\mu }{2}\left( \mathrm {tr}(\overline{\varvec{C}}) - 3\right) + \frac{\kappa }{2} {(J-1)}^2, \end{aligned}$$

with parameters $\mu =5704.7\,\hbox {kPa}$ and $\kappa =283{,}333\,\hbox {kPa}$ for the nearly incompressible and $1/\kappa =0$ for the truly incompressible case. For the results presented, we considered hexahedral and tetrahedral meshes with five levels of refinement, respectively; for discretization details of the column meshes see Table 4. In Fig. 15, mesh convergence with respect to tip displacement $(u_x,u_y,u_z)$ at point **D** is analyzed. While differences at lower levels of refinement $\ell =1,2$ are severe, the displacements converge for higher levels of refinement $\ell =3,4,5$. For finer grids the curves for tetrahedral and hexahedral elements are almost indistinguishable, see also Fig. 16, and the results are in good agreement with those presented in [71]. While this figure was produced using MINI elements we also observed a similar behavior of mesh convergence for the projection-based stabilization. In fact, for the finest grid, all the proposed stabilization techniques and elements gave virtually identical results, see Fig. 16. Further, as already observed by Scovazzi et al. [71], the fully and nearly incompressible formulations gave almost identical deformations, see Fig. 17.

**Table 4:** Properties of *column meshes* used in Sect. 4.4

Hexahedral meshes			Tetrahedral meshes		
$\ell$	Elements	Nodes	$\ell$	Elements	Nodes
1	48	117	1	240	117
2	384	625	2	1920	625
3	3072	3969	3	15,360	3969
4	24,576	28,033	4	122,880	28,033
5	196,608	210,177	5	983,040	210,177

**Fig. 18:**
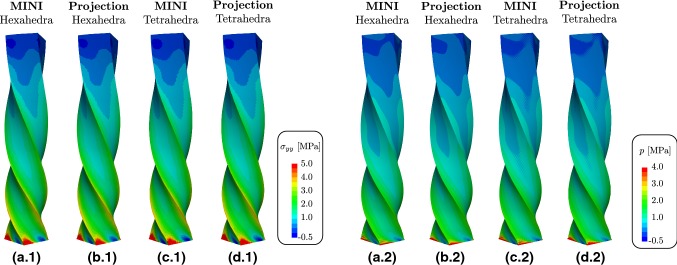
*Twisting column test:***a** stress $\sigma _{yy}$ and **b** hydrostatic pressure *p* contours at time instant $t=0.3\,\hbox {s}$ for the different grids and stabilization techniques

**Fig. 19:**
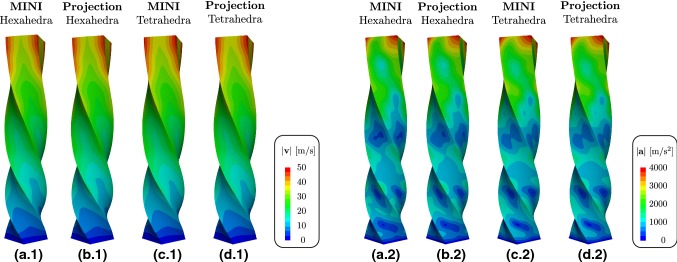
*Twisting column test:* magnitude of **a** velocity ${\MakeLowercase {\mathbf {v}}}$ and **b** acceleration ${\MakeLowercase {\mathbf {a}}}$ at time instant $t=0.3\,\hbox {s}$ for the different grids and stabilization techniques

In Fig. 18 stress $\sigma _{yy}$ and pressure *p* contours are plotted on the deformed configuration for the incompressible case at time instant $t=0.3\,\hbox {s}$. Minor pressure oscillations can be observed for tetrahedral elements. Again, results match well those presented in [71, Figure 22].

Finally, in Fig. 19, we compare the magnitude of velocity and acceleration at time instant $t=0.3\,\hbox {s}$. Results for these variables are very smooth and hardly distinguishable for all the different approaches.

The *computational costs* for this nonlinear elasticity problem were significant due to the required solution of a saddle-point problem in each Newton step and a large number of time steps. However, this challenge can be addressed by using a massively parallel iterative solving method and exploiting potential of modern HPC hardware. The most expensive simulations were the fully incompressible cases for the finest grids with a total of 840,708 degrees of freedom and 400 time steps. These computations were executed at the national HPC computing facility ARCHER in the United Kingdom using 96 cores. Computational times were as follows: $239\,\hbox {min}$ for tetrahedral meshes and projection-based stabilization; $283\,\hbox {min}$ for tetrahedral meshes and MINI elements; $449\,\hbox {min}$ for hexahedral meshes and projection-based stabilization; and $752.5\,\hbox {min}$ for hexahedral meshes and MINI elements. Simulation times for nearly incompressible problems were lower, ranging from 177 to $492\,\hbox {min}$. This is due to the additional matrix on the lower-right side of the block stiffness matrix which led to a smaller number of linear iterations. Simulations with hexahedral meshes were, in general, computationally more expensive compared to simulations with tetrahedral grids; the reason being mainly a higher number of linear iterations. Computational burden for MINI elements was larger due to higher matrix assembly times. However, this assembly time is highly scalable as there is almost no communication cost involved in this process.

## Conclusion

In this study we described methodology for modeling nearly and fully incompressible solid mechanics for a large variety of different scenarios. A stable MINI element was presented which can serve as an excellent choice for applied problems where the use of higher order element types is not desired, e.g., due to fitting accuracy of the problem domain. We also proposed an easily implementable and computationally cheap technique based on a local pressure projection. Both approaches can be applied to stationary as well as transient problems without modifications and perform excellent with both hexahedral and tetrahedral grids. Both approaches allow a straightforward inclusion in combination with existing finite element codes since all required implementations are purely on the element level and are well-suited for simple single-core simulations as well as HPC computing. Numerical results demonstrate the robustness of the formulations, exhibiting a great accuracy for selected benchmark problems from the literature.

While the proposed projection method works well for relatively stiff materials as considered in this paper, the setting of the parameter $\mu ^*$ has to be adjusted for soft materials such as biological tissues. A further limitation is that both formulations render the need of solving a block system, which is computationally more demanding and suitable preconditioning is not trivial. However, the MINI element approach can be used without further tweaking of artificial stabilization coefficients and preliminary results suggested robustness, even for very soft materials. Consistent linearization as presented ensures that quadratic convergence of the Newton–Raphson algorithm was achieved for all the problems considered. Note that all computations for forming the tangent matrices and also the right hand side residual vectors are kept local to each element. This benefits scaling properties of parallel codes and also enables seamless implementation in standard finite element software.

The excellent performance of the methods along with their high versatility ensure that this framework serves as a solid platform for simulating nearly and fully incompressible phenomena in stationary and transient solid mechanics. In future studies, we plan to extend the formulation to anisotropic materials with stiff fibers as they appear for example in the simulation of cardiac tissue and arterial walls.

## Appendix

### Generalized-$\alpha$ time integration

After spatial discretization of (46) and (47) we get the following degenerate hyperbolic system

$$\begin{aligned} \rho _0\varvec{M}_h \ddot{{\MakeLowercase {\mathbf {u}}}}(t) + \varvec{C}_h \dot{{\MakeLowercase {\mathbf {u}}}}(t) + \varvec{\mathbf {R}}_\mathrm {upper}({\MakeLowercase {\mathbf {u}}}(t), {\MakeLowercase {\mathbf {p}}}(t))&= {\MakeLowercase {\mathbf {0}}},\\ \varvec{\mathbf {R}}_\mathrm {lower}({\MakeLowercase {\mathbf {u}}}(t), {\MakeLowercase {\mathbf {p}}}(t))&= {\MakeLowercase {\mathbf {0}}},\\ {\MakeLowercase {\mathbf {u}}}(0)&= {\MakeLowercase {\mathbf {u}}}_0,\\ \dot{{\MakeLowercase {\mathbf {u}}}}(0)&= {\MakeLowercase {\mathbf {u}}}_0, \end{aligned}$$

where $\varvec{M}_h$ denotes the mass matrix; $\varvec{C}_h$ denotes an optional damping matrix; $\ddot{{\MakeLowercase {\mathbf {u}}}}(t)$ denote the unknown nodal accelerations; $\dot{{\MakeLowercase {\mathbf {u}}}}(t)$ denote the unknown nodal velocities; ${\MakeLowercase {\mathbf {u}}}(t)$ denote the unknown nodal displacements; and ${\MakeLowercase {\mathbf {p}}}(t)$ denote the unknown nodal pressure values. We will use the modified generalized-$\alpha$ method proposed in [50]. To this end we introduce the auxiliary velocity ${\MakeLowercase {\mathbf {v}}} = \dot{{\MakeLowercase {\mathbf {u}}}}$. Then, applying the standard generalized-$\alpha$ integrator from [28] we obtain

48 $$\begin{aligned}&\varvec{M}_h \dot{{\MakeLowercase {\mathbf {u}}}}_{n+\alpha _\mathrm {m}} - \varvec{M}_h {\MakeLowercase {\mathbf {v}}}_{n+\alpha _\mathrm {f}} = {\MakeLowercase {\mathbf {0}}}, \end{aligned}$$

49 $$\begin{aligned}&\rho _0 \varvec{M}_h \dot{{\MakeLowercase {\mathbf {v}}}}_{n+\alpha _\mathrm {m}} + \varvec{C}_h {\MakeLowercase {\mathbf {v}}}_{n+\alpha _\mathrm {f}} + \varvec{\mathbf {R}}_\mathrm {upper}^{n+\alpha _\mathrm {f}} = {\MakeLowercase {\mathbf {0}}},\end{aligned}$$

50 $$\begin{aligned}&\varvec{\mathbf {R}}_\mathrm {lower}^{n+\alpha _\mathrm {f}} = {\MakeLowercase {\mathbf {0}}}, \end{aligned}$$

where

$$\begin{aligned} \varvec{\mathbf {R}}_\mathrm {upper}^{n+\alpha _\mathrm {f}}&:= \alpha _\mathrm {f} \varvec{\mathbf {R}}_\mathrm {upper}({\MakeLowercase {\mathbf {u}}}_{n+1}, {\MakeLowercase {\mathbf {p}}}_{n+1}),\\&\qquad +\,(1-\alpha _\mathrm {f})\varvec{\mathbf {R}}_\mathrm {upper}({\MakeLowercase {\mathbf {u}}}_{n}, {\MakeLowercase {\mathbf {p}}}_{n}),\\ \varvec{\mathbf {R}}_\mathrm {lower}^{n+\alpha _\mathrm {f}}&:= \alpha _\mathrm {f} \varvec{\mathbf {R}}_\mathrm {lower}({\MakeLowercase {\mathbf {u}}}_{n+1}, {\MakeLowercase {\mathbf {p}}}_{n+1}),\\&\qquad +\,(1-\alpha _\mathrm {f})\varvec{\mathbf {R}}_\mathrm {lower}({\MakeLowercase {\mathbf {d}}}_{n}, {\MakeLowercase {\mathbf {p}}}_{n}), \end{aligned}$$

and

51 $$\begin{aligned} \dot{{\MakeLowercase {\mathbf {u}}}}_{n+\alpha _\mathrm {m}}&:= \alpha _\mathrm {m}\dot{{\MakeLowercase {\mathbf {u}}}}_{n+1} + (1-\alpha _\mathrm {m}) \dot{{\MakeLowercase {\mathbf {u}}}}_n, \end{aligned}$$

52 $$\begin{aligned} \dot{{\MakeLowercase {\mathbf {v}}}}_{n+\alpha _\mathrm {m}}&:= \alpha _\mathrm {m}\dot{{\MakeLowercase {\mathbf {v}}}}_{n+1} + (1-\alpha _\mathrm {m}) \dot{{\MakeLowercase {\mathbf {v}}}}_n,\end{aligned}$$

53 $$\begin{aligned} {\MakeLowercase {\mathbf {v}}}_{n+\alpha _\mathrm {f}}&:= \alpha _\mathrm {f} {\MakeLowercase {\mathbf {v}}}_{n+1} + (1-\alpha _\mathrm {f}) \ {\MakeLowercase {\mathbf {v}}}_n. \end{aligned}$$

Moreover, we employ Newmark’s approximations, [61],

54 $$\begin{aligned} \dot{{\MakeLowercase {\mathbf {u}}}}_{n+1}&= \frac{1}{\gamma \varDelta t}\left( {\MakeLowercase {\mathbf {u}}}_{n+1} - {\MakeLowercase {\mathbf {u}}}_{n}\right) + \frac{\gamma -1}{\gamma }\dot{{\MakeLowercase {\mathbf {u}}}}_n, \end{aligned}$$

55 $$\begin{aligned} {\MakeLowercase {\mathbf {v}}}_{n+1}&= \frac{1}{\gamma \varDelta t}\left( {\MakeLowercase {\mathbf {v}}}_{n+1} - {\MakeLowercase {\mathbf {v}}}_n\right) + \frac{\gamma -1}{\gamma } \dot{{\MakeLowercase {\mathbf {v}}}}_n \end{aligned}$$

Using (48) we observe

$$\begin{aligned} \dot{{\MakeLowercase {\mathbf {u}}}}_{n+\alpha _\mathrm {m}} = {\MakeLowercase {\mathbf {v}}}_{n+\alpha _\mathrm {f}} \end{aligned}$$

and combining this with (49)–(55) we conclude

$$\begin{aligned} {\MakeLowercase {\mathbf {v}}}_{n+1}&= \frac{\alpha _\mathrm {m}}{\alpha _\mathrm {f} \gamma \varDelta t}\left( {\MakeLowercase {\mathbf {u}}}_{n+1} - {\MakeLowercase {\mathbf {u}}}_n\right) + \frac{\gamma - \alpha _\mathrm {m}}{\gamma \alpha _\mathrm {f}} \dot{{\MakeLowercase {\mathbf {u}}}}_n + \frac{\alpha _\mathrm {f}-1}{\alpha _\mathrm {f}} {\MakeLowercase {\mathbf {v}}}_n,\\ \dot{{\MakeLowercase {\mathbf {v}}}}_{n+1}&= \frac{\alpha _\mathrm {m}}{\alpha _\mathrm {f} \gamma ^2 \varDelta t^2}\left( {\MakeLowercase {\mathbf {u}}}_{n+1}- {\MakeLowercase {\mathbf {u}}}_n\right) - \frac{1}{\alpha _\mathrm {f} \gamma \varDelta t} {\MakeLowercase {\mathbf {v}}}_n + \frac{\gamma -1}{\gamma } \dot{{\MakeLowercase {\mathbf {v}}}}_n\\&\quad +\,\frac{\gamma - \alpha _\mathrm {m}}{\alpha _\mathrm {f} \gamma ^2 \varDelta t} \dot{{\MakeLowercase {\mathbf {u}}}}_n. \end{aligned}$$

Thus, a dependence of ${\MakeLowercase {\mathbf {v}}}_{n+1}$ and $\dot{{\MakeLowercase {\mathbf {v}}}}_{n+1}$ on ${\MakeLowercase {\mathbf {u}}}_{n+1}$ can be established. Having this the unknown values ${\MakeLowercase {\mathbf {u}}}_{n+1}, {\MakeLowercase {\mathbf {p}}}_{n+1}$ can be computed with the Newton–Raphson method. Based on [50] we set the parameters depending only on $\rho _\infty \in [0,1)$ by

$$\begin{aligned} \alpha _\mathrm {f}&:= \frac{1}{1 + \rho _\infty },\\ \alpha _\mathrm {m}&:= \frac{3 - \rho _\infty }{2(1 + \rho _\infty )},\\ \gamma&:= \frac{1}{2}+\alpha _\mathrm {m}-\alpha _\mathrm {f}. \end{aligned}$$

In all our simulations we used a value of $\rho _\infty = 0.5$.

### Remark on the implementation of the pressure-projection stabilized equal order pair

Considering the bilinear form $s_h(p_h, q_h)$ defined in (38) we can rewrite this with a simple calculation into

$$\begin{aligned} s_h(p_h, q_h) := \sum _{l=1}^{n_\mathrm {el}}\left( \int \limits _{K_l} p_h q_h\,\mathrm {d}{\MakeLowercase {\mathbf {x}}}- \frac{1}{|\tau _l|}\int \limits _{K_l} p_h \,\mathrm {d}{\MakeLowercase {\mathbf {x}}}\int \limits _{K_l}q_h\,\mathrm {d}{\MakeLowercase {\mathbf {x}}}\right) . \end{aligned}$$

Denoting by $\{\phi _i\}_{i=1}^n$ the chosen ansatz functions the element contribution for an arbitrary element *K* to the matrix $\varvec{C}_h$ is given by

$$\begin{aligned} \int \limits _{K} \phi _i \phi _j \,\mathrm {d}{\MakeLowercase {\mathbf {x}}}- \frac{1}{|K |} \int \limits _{K} \phi _i \,\mathrm {d}{\MakeLowercase {\mathbf {x}}}\int \limits _{K} \phi _j \,\mathrm {d}{\MakeLowercase {\mathbf {x}}}. \end{aligned}$$

This corresponds to an element mass matrix minus a rank-one correction.

### Static condensation

For completeness we provide a summary for the static condensation used for the MINI element. Consider a finite element $K \in \mathcal {T}_h$ with a local ordering of the unknowns ${\MakeLowercase {\mathbf {u}}}$

$$\begin{aligned}&{\MakeLowercase {\mathbf {u}}} = \left( u_x^1, u_y^1, u_z^1, \ldots , u_x^{\mathrm {ndofs}_N}, u_y^{\mathrm {ndofs}_\mathrm {N}}, u_z^{\mathrm {ndofs}_\mathrm {N}}, \right. \\&\qquad \left. u_{x,\mathrm {B}}^1, u_{y,\mathrm {B}}^1, u_{z,\mathrm {B}}^1, \ldots , u_{x,\mathrm {B}}^{\mathrm {ndofs}_\mathrm {B}}, u_{y,\mathrm {B}}^{\mathrm {ndofs}_\mathrm {B}}, u_{z,\mathrm {B}}^{\mathrm {ndofs}_\mathrm {B}}\right) \end{aligned}$$

and $${\MakeLowercase {\mathbf {p}}}$$ as

$$\begin{aligned} {\MakeLowercase {\mathbf {p}}} = \left( p^1,p^2,\ldots ,p^{\mathrm {ndofs}_\mathrm {N}}\right) . \end{aligned}$$

Here, $\mathrm {ndofs}_\mathrm {N}$ corresponds to the nodal degrees of freedom per element and $\mathrm {ndofs}_\mathrm {B}$ to the bubble degrees of freedom (one for tetrahedral elements and two for hexahedral elements). Then the element contribution to the global saddle-point system can be written as

$$\begin{aligned} \begin{pmatrix} \varvec{K}_\mathrm {NN} &{}\quad \varvec{K}_\mathrm {NB} &{}\quad \varvec{B}_\mathrm {N}^\top \\ \varvec{K}_\mathrm {BN} &{}\quad \varvec{K}_\mathrm {BB} &{}\quad \varvec{B}_\mathrm {B}^\top \\ \varvec{B}_\mathrm {N} &{}\quad \varvec{B}_\mathrm {B} &{}\quad \varvec{C}_\mathrm {N} \end{pmatrix} \begin{pmatrix} \varDelta {\MakeLowercase {\mathbf {u}}}_\mathrm {N}\\ \varDelta {\MakeLowercase {\mathbf {u}}}_\mathrm {B}\\ \varDelta {\MakeLowercase {\mathbf {p}}} \end{pmatrix} = \begin{pmatrix} -\varvec{\mathbf {R}}_\mathrm {N}^\mathrm {upper}\\ -\varvec{\mathbf {R}}_\mathrm {B}^\mathrm {upper}\\ -\varvec{\mathbf {R}}_\mathrm {N}^\mathrm {lower} \end{pmatrix} . \end{aligned}$$

The bubble part of the stiffness matrix, $\varvec{K}_\mathrm {BB}$ is local to the element and can be directly inverted. This gives the condensed system

$$\begin{aligned} \begin{pmatrix} \varvec{K}_\mathrm {eff} &{}\quad \varvec{B}_\mathrm {eff}^T \\ \varvec{B}_\mathrm {eff} &{}\quad \varvec{C}_\mathrm {eff} \end{pmatrix} \begin{pmatrix} \varDelta {\MakeLowercase {\mathbf {u}}}_\mathrm {N}\\ \varDelta {\MakeLowercase {\mathbf {p}}} \end{pmatrix} = \begin{pmatrix} -\varvec{\mathbf {R}}_\mathrm {eff}^\mathrm {upper}\\ -\varvec{\mathbf {R}}_\mathrm {eff}^\mathrm {lower} \end{pmatrix} , \end{aligned}$$

where the effective matrices and vectors are given as

$$\begin{aligned} \varvec{K}_\mathrm {eff}&:= \varvec{K}_\mathrm {NN} - \varvec{K}_\mathrm {NB}\varvec{K}_\mathrm {BB}^{-1}\varvec{K}_\mathrm {BN}\\ \varvec{B}_\mathrm {eff}&:= \varvec{B}_\mathrm {N} - \varvec{B}_\mathrm {B}\varvec{K}_\mathrm {BB}^{-1}\varvec{K}_\mathrm {BN},\\ \varvec{C}_\mathrm {eff}&:= \varvec{C}_\mathrm {N} - \varvec{B}_\mathrm {B}\varvec{K}_\mathrm {BB}^{-1}\varvec{B}_\mathrm {B}^\top ,\\ \varvec{\mathbf {R}}_\mathrm {eff}^\mathrm {upper}&:= \varvec{\mathbf {R}}_\mathrm {N}^\mathrm {upper} - \varvec{K}_\mathrm {NB} \varvec{K}_\mathrm {BB}^{-1} \varvec{\mathbf {R}}_\mathrm {B}^\mathrm {upper},\\ \varvec{\mathbf {R}}_\mathrm {eff}^\mathrm {lower}&:= \varvec{\mathbf {R}}_\mathrm {N}^\mathrm {lower} - \varvec{B}_\mathrm {B} \varvec{K}_\mathrm {BB}^{-1} \varvec{\mathbf {R}}_\mathrm {B}^\mathrm {upper}. \end{aligned}$$

The effective matrices and vectors can then be assembled in a standard way into the global system. The bubble update contributions can be calculated once $\varDelta {\MakeLowercase {\mathbf {u}}}_N$ and $\varDelta {\MakeLowercase {\mathbf {p}}}_N$ are know as

$$\begin{aligned} \varDelta {\MakeLowercase {\mathbf {u}}}_B = -\varvec{K}_\mathrm {BB}^{-1} \left( \varvec{\mathbf {R}}_\mathrm {B}^\mathrm {upper} + \varvec{K}_\mathrm {BN} \varDelta {\MakeLowercase {\mathbf {u}}}_\mathrm {N} + \varvec{B}_\mathrm {B}^\top \varDelta {\MakeLowercase {\mathbf {p}}}_\mathrm {N}\right) . \end{aligned}$$

### Tensor calculus

We use the following results from tensor calculus, for more details we refer to, e.g., [45, 84].

$$\begin{aligned} \frac{\partial \overline{\varvec{C}}}{\partial \varvec{C}}&= J^{-\frac{2}{3}} \mathbb {P} = J^{-\frac{2}{3}} \left( \mathbb {I} - \frac{1}{3}\varvec{C}^{-1} \otimes \varvec{C}\right) ,\\ \frac{\partial \varvec{C}^{-1}}{\partial \varvec{C}}&= - \varvec{C}^{-1} \odot \varvec{C}^{-1},\\ {(\varvec{A}\odot \varvec{A})}_{ijkl}&:= \frac{1}{2}\left( A_{ik}A_{jl}+A_{il}A_{jk}\right) . \end{aligned}$$

For symmetric $\varvec{A}$ it holds

$$\begin{aligned} \mathbb {P} : \varvec{A} = \mathrm {Dev}(\varvec{A}) = \varvec{A} - \frac{1}{3}(\varvec{A} : \varvec{C}) \varvec{C}^{-1}. \end{aligned}$$

The isochoric part of the second Piola–Kirchhoff stress tensor as well as the isochoric part of the fourth order elasticity tensor are given as

56 $$\begin{aligned} \varvec{S}_\mathrm {isc}&:= 2 \frac{\partial \overline{\varPsi }(\overline{\varvec{C}})}{\partial \varvec{C}} = J^{-\frac{2}{3}}\mathrm {Dev}(\overline{\varvec{S}}),\nonumber \\ \overline{\varvec{S}}&:= 2 \frac{\partial \overline{\varPsi }(\overline{\varvec{C}})}{\partial \overline{\varvec{C}}}, \end{aligned}$$

57 $$\begin{aligned} \mathbb {C}_\mathrm {isc}&:= 4 \frac{\overline{\varPsi }(\overline{\varvec{C}})}{\partial \varvec{C} \partial \varvec{C}} \nonumber \\&= J^{-\frac{4}{3}} \mathbb {P} \overline{\mathbb {C}} \mathbb {P}^\top + J^{-\frac{2}{3}} \frac{2}{3}\mathrm {tr}(\varvec{C} \overline{\varvec{S}}) \widetilde{\mathbb {P}}\nonumber \\&\quad -\, \frac{4}{3}\varvec{S}_\mathrm {isc} \overset{\mathrm {S}}{\otimes }\varvec{C}^{-1},\nonumber \\ \overline{\mathbb {C}}&:= 4\frac{\partial \overline{\varPsi } (\overline{\varvec{C}})}{\partial \overline{\varvec{C}} \partial \overline{\varvec{C}}},\nonumber \\ \widetilde{\mathbb {P}}&:= \varvec{C}^{-1} \odot \varvec{C}^{-1} - \frac{1}{3} \varvec{C}^{-1} \otimes \varvec{C}^{-1},\nonumber \\ \varvec{A}\overset{\mathrm {S}}{\otimes }\varvec{B}&:= \frac{1}{2}\left( \varvec{A}\otimes \varvec{B} + \varvec{B} \otimes \varvec{A} \right) . \end{aligned}$$

## Footnotes

1 https://www.mcs.anl.gov/petsc/petsc-current/docs/manualpages/PC/PCFIELDSPLIT.html.

2 https://www.mcs.anl.gov/petsc/petsc-current/docs/manualpages/Mat/MatGetInfo.html.
